# A multicomponent complex intervention for supportive follow-up of persons with chronic heart failure: a randomized controlled pilot study (the UTILE project)

**DOI:** 10.1186/s40814-023-01338-7

**Published:** 2023-06-27

**Authors:** Petra Schäfer-Keller, Denis Graf, Kris Denhaerynck, Gabrielle Cécile Santos, Josepha Girard, Marie-Elise Verga, Kelly Tschann, Grégoire Menoud, Anne-Laure Kaufmann, Marcia Leventhal, David A. Richards, Anna Strömberg

**Affiliations:** 1 Institute of Applied Research in Health, School of Health Sciences Fribourg, HES-SO University of Applied Sciences and Arts Western Switzerland, Fribourg, Switzerland; 2 Cardiology, HFR Fribourg - Hôpital Cantonal, Fribourg, Switzerland; 3 Institute of Nursing Science, Department of Public Health, University of Basel, Basel, Switzerland; 4 Data Acquisition Unit, HES-SO Valais-Wallis, University of Applied Sciences and Arts Western Switzerland, Sion, Switzerland; 5 Present address: Pfeffingen, Switzerland; 6 Faculty of Health and Life Sciences, University of Exeter, Exeter, UK; 7 Department of Health and Caring Sciences, Western Norway University of Applied Sciences, Bergen, Norway; 8 Department of Health, Medicine and Caring Sciences and Department of Cardiology, Linköping University, Linköping, Sweden

**Keywords:** Heart failure, Nursing, Supportive follow-up, Multicomponent complex intervention, Feasibility, Acceptability, Pilot randomized controlled trial

## Abstract

**Background:**

Heart failure (HF) is a progressive disease associated with a high burden of symptoms, high morbidity and mortality, and low quality of life (QoL). This study aimed to evaluate the feasibility and potential outcomes of a novel multicomponent complex intervention, to inform a future full-scale randomized controlled trial (RCT) in Switzerland.

**Methods:**

We conducted a pilot RCT at a secondary care hospital for people with HF hospitalized due to decompensated HF or with a history of HF decompensation over the past 6 months. We randomized 1:1; usual care for the control (CG) and intervention group (IG) who received the intervention as well as usual care. Feasibility measures included patient recruitment rate, study nurse time, study attrition, the number and duration of consultations, intervention acceptability and intervention fidelity. Patient-reported outcomes included HF-specific self-care and HF-related health status (KCCQ-12) at 3 months follow-up. Clinical outcomes were all-cause mortality, hospitalization and days spent in hospital.

**Results:**

We recruited 60 persons with HF (age mean = 75.7 years, ± 8.9) over a 62-week period, requiring 1011 h of study nurse time. Recruitment rate was 46.15%; study attrition rate was 31.7%. Follow-up included 2.14 (mean, ± 0.97) visits per patient lasting a total of 166.96 min (mean, ± 72.55), and 3.1 (mean, ± 1.7) additional telephone contacts. Intervention acceptability was high. Mean intervention fidelity was 0.71. We found a 20-point difference in mean self-care management change from baseline to 3 months in favour of the IG (Cohens’ *d* = 0.59). Small effect sizes for KCCQ-12 variables; less IG participants worsened in health status compared to CG participants. Five deaths occurred (IG = 3, CG = 2). There were 13 (IG) and 18 (CG) all-cause hospital admissions; participants spent 8.90 (median, IQR = 9.70, IG) and 15.38 (median, IQR = 18.41, CG) days in hospital. A subsequent full-scale effectiveness trial would require 304 (for a mono-centric trial) and 751 participants (for a ten-centre trial) for HF-related QoL (effect size = 0.3; power = 0.80, alpha = 0.05).

**Conclusion:**

We found the intervention, research methods and outcomes were feasible and acceptable. We propose increasing intervention fidelity strategies for a full-scale trial.

**Trial registration:**

ISRCTN10151805, retrospectively registered 04/10/2019.

**Supplementary Information:**

The online version contains supplementary material available at 10.1186/s40814-023-01338-7.

## Key messages regarding feasibility

- What uncertainties existed regarding feasibility
  - Feasibility of recruiting and retaining persons with HF for a randomized controlled trial testing the effectiveness of a novel multicomponent complex intervention
  - Feasibility for delivering the multicomponent complex intervention
  - Acceptability of receiving the multicomponent complex intervention
  - Outcome responsiveness of the multicomponent complex intervention on patient-reported and clinical outcomes; unknown effect sizes for relevant variables for sample size calculation for a future fully powered randomized controlled trial
- What are the key findings?
  - 46% patient recruitment rate; 31% patient attrition rate
  - 0.71 intervention fidelity across all components and deliveries
  - High intervention acceptability across all dimensions, lowest scores for perceived study participation burden
  - Outcome responsiveness for HF-specific self-care, HF-related health status, hospital admissions and days spent in hospital, small to medium effect sizes
- What are the implications of the findings for the design of a future full-scale study?
  - A demanding recruitment process, requesting research nurses to dedicate sufficient working hours for recruitment
  - Requires strong commitments of cardiologists for eligibility screening of HF diagnosis, especially when recruitment occurs in general wards
  - Engaging in Patient and Public Involvement for identifying options for decreasing the burden of trial participation for study participants
  - Enhancing intervention fidelity strategies alongside delivering the multicomponent complex intervention
  - Update the multicomponent complex intervention in line with new treatment guidelines, but no significant amendments for the intervention required
  - Intervention effectiveness testing requires a mono-centre trial at a large clinical facility or a multicentre trial

## Background and objectives

Heart failure (HF) is a major health concern associated with high mortality and morbidity, frequent hospital admissions and low quality of life [1]. Globally, its prevalence is estimated at 5–9% in individuals aged 65 or older [2–4], and continues to rise [1]. While treatments have improved in recent years, all-cause mortality and hospitalization rates remain high [2]. Reducing hospitalizations and mortality, as well as improving clinical status, functional capacity and quality of life, are high-priority objectives for this population [1].

To improve outcomes, the European Society of Cardiology (ESC) [1] recommends that all persons with HF should receive a multiple component care package. Such care should be person-/patient-centred and have a holistic approach, be tailored to individual needs, and be delivered by competent health care professionals. Focusing solely on HF management, or providing patient education alone, have each been shown to be ineffective in improving well-being and clinical outcomes [1].

Internationally, various multidisciplinary disease-management programmes [5, 6] have been tested in HF, demonstrating effectiveness on all-cause mortality, morbidity and quality of life. The content and structure of programmes vary between studies and health care settings; the best results were associated with nurse-led programmes, including telephone follow-up [6] and/or home visits by a nurse [5]. Also, self-care intervention studies have demonstrated beneficial effects [7]. Few studies have provided clear descriptions of the interventions, thus impairing their replication [8–10]. In Switzerland, research related to such types of interventions including HF nurses is scarce [11, 12]. Therefore, a fully powered randomized controlled trial is required in Swiss contexts to evaluate the effectiveness of such an intervention [13]. First, however, it is necessary to address the methodological and procedural uncertainties associated with such a trial, including a lack of appropriate data to estimate the intervention’s effect size, as well as the intervention’s acceptability within the target context [14].

Persons with HF clearly benefit from multidisciplinary disease-management programmes [1]. Specifically, the 2016 ESC guidelines recommended a multicomponent follow-up [2]. We developed and tested a multicomponent complex intervention for the supportive follow-up of persons with HF, guided by the Medical Research Council (MRC) framework for the development and evaluation of complex interventions in health [13–18]. Our intervention is informed by relevant literature [5–7, 9, 19–22] and the results of several small-scale descriptive studies conducted in the context of a partnership between our university of applied science and a secondary care hospital [23–26].

The primary objective of this pilot randomized controlled trial (RCT) was to test the feasibility of a novel nurse-delivered multicomponent complex intervention for the supportive follow-up of persons with chronic HF (hereafter, “intervention”). A secondary objective was to provide information on patient-reported and clinical outcomes to inform the design of a future fully powered RCT investigating the effectiveness of the intervention in the Swiss context.

## Methods

### Aim

We aimed to assess the following: patient recruitment and participant retention over the 3-month follow-up period; the number of delivered interventions in clinic or at home; intervention duration; fidelity to the intervention; intervention acceptability; and to explore the intervention’s potential effect on patient-reported and clinical outcomes.

### Design

We undertook a single-centre, pragmatic, two-arm 1:1 randomized, parallel pilot RCT including an embedded concurrent process study using quantitative data on patient recruitment and retention to assess feasibility, and a qualitative study exploring the acceptability of the intervention and trial procedures from the perspectives of persons with HF, physicians and nurses. We will report the results of the qualitative study elsewhere. This paper is published according to the Extension of CONSORT to pilot trials [27] and according to the CONSERVE 2021 Statement [28] given that the onset of the COVID-19 pandemic occurred during the study.

### Participants

We recruited adults with HF (≥ 18 years of age) with the following inclusion criteria: (a) diagnosed HF with reduced, mildly reduced or preserved ejection fraction in New York Heart Association (NYHA) functional classes II‐IV; (b) hospitalized in the internal medicine departments of two campuses of one hospital; (c) reason for current hospitalization either decompensated HF or other reasons but with a history of hospitalization within the past 6 months due to decompensated HF. Other conditions were to provide written informed consent, and speaking French or German. We excluded persons with HF with (a) any inability to follow the procedures of the study (due to language problems, psychological disorders, cognitive impairment), (b) who suffered from immediately life-threatening illness or (c) with short expected survival, dementia or serious comorbidities or complications (e.g. untreated psychiatric illness, untreated malignancies). We also excluded patients with COVID-19, positive test for SARS-CoV2, or a positive anamnesis regarding SARS-CoV2 infection while waiting for test results.

### Settings and locations where the data were collected

We conducted the study at one campus of a non-university hospital providing regional secondary care for persons with HF in internal medicine and the cardiology outpatient department. During the trial, a second campus of the same hospital was added to accelerate recruitment, which had been an option put forward in the protocol in case of low recruitment progress (ISRCTN101518059). However, the opening of the second campus occurred during the onset of the COVID-19 pandemic, which eventually limited staff availability and access to the site. Thus, recruitment occurred predominantly at the initial campus.

### Interventions

#### Control condition

Control group (CG) care included standard in-hospital care as well as post-discharge follow-up care by general practitioners (GP) and cardiologists. During the inpatient phase, participants received the Swiss Heart Foundation’s “Heart Failure Patient Kit” (a printed information pack) in French or German [29] from the research nurse. A cardiology nurse (in the first campus) or a ward nurse (in the second campus) provided HF patient education during the inpatient phase or shortly thereafter during a follow-up meeting focusing on self-care skills. Patient education occurred during one or two face-to-face encounters and before discharge from hospital. Knowledge acquisition and development was facilitated via motivational interviewing communication techniques [30, 31], for which cardiology and ward nurses in routine care had been previously trained within their Bachelor of Nursing training and/or advanced studies in cardiology.

#### Intervention condition

The intervention (Table 1) was specifically designed for this study. It is based on the 2016 ESC recommendations for supportive follow-up of persons with HF [2] and also in line with recommendations in the current 2021 ESC guidelines [1], the middle-range theory of self-care in chronic disease [32], the situation-specific theory of HF self-care [33, 34], and the results of needs assessment studies in our context [23–26]. The intervention aims at preventing cardiac decompensation and delaying HF progression. It is composed of (1) patient involvement in symptom monitoring and support for self-care capabilities; (2) facilitation of early decompensation detection; (3) optimized medical and device treatment following ESC guidelines; (4) psychosocial support for patient and family; (5) patient education; (6) easy access to care; and (7) facilitation of multidisciplinary collaboration [2]. We operationalized these components for nurse delivery [23].

**Table 1:** Intervention description

Item TIDieR	Intervention description
**Brief name**	**Provide the name or a phrase that describes the intervention** A multicomponent complex nurse-delivered intervention informed by the European Society of Cardiology guidelines for a supportive follow-up of persons with heart failure
**Why**	**Describe any rationale, theory, or goal of the elements essential to the intervention** - The 2016 European Society of Cardiology guidelines [2] recommend a structured multicomponent supportive follow-up in HF including (a) patients’ symptom monitoring and self-care capabilities support; (b) early detection of impending decompensation; (c) optimized medical and device treatment; (d) patient self-care education; (e) psychosocial support for patients and families; (f) facilitated access to care; and (g) multidisciplinary collaboration - Small-scale studies conducted to define the problem and determine the needs within our context showed, that ○ Physicians and nurses reported important barriers to patient-centred care but felt a strong need to provide it [24] ▪ Goal: use a patient-centred approach ○ Persons with HF reported a high prevalence of inadequate self-care on virtually all relevant items, while showing important clinical characteristics that would potentially limit their self-care capabilities [25] ▪ Goal: considering self-care capabilities alongside patients’ vulnerability characteristics ○ Nurse-provided HF patient education rarely addressed individuals’ self-care levels and barriers to self-care as well as nurses reported a lack of appropriate time or role to perform adequate patient self-care education [26] ▪ Goal: considering self-care capabilities to make patient self-care education meaningful; considering providing nurse support for, e.g. symptom stability ▪ Goal: offering several follow-up visits and on a needs-led basis - Central foci /priorities from a range of patients’ self-care capabilities ○ Symptom management: in our previous study on this topic, only 10% of patients reported adequate responses when experiencing dyspnoea or peripheral oedema, while 61% reported confidence in their abilities to react appropriately when symptoms occurred (implication: perceived self-efficacy does not reliably reflect patients’ actual symptom management abilities as they arise) [25] ○ Medication adherence: medication non-adherence jeopardizes outcomes [2] ○ Physical activity: regular aerobic exercise improves functional capacity and symptoms and improves outcomes in persons with HF [2] - Combination of counselling, care and treatment [19]: ○ Assessment of patients’ self-care capabilities ○ Assessment of the patients’ health status, symptom experience, and barriers to self-care
	Goal: to provide standardized, tailored self-care education and symptom management support, to support self-care capabilities, to facilitate multidisciplinary collaboration to improve negative outcomes
**What materials**	**Materials: Describe any physical or informational materials used in the intervention, including those provided to participants or used in intervention delivery or in training of intervention providers. Provide information on where the materials can be accessed (e.g. online appendix, URL)** - Intervention manual - French or German version of the “Heart Failure Patient kit” from the Swiss Heart Foundation by a research assistant - Web-based self-report tool (see below for description) access via tablet - Vscan Extend Dual Wi-Fi portable ultrasound device - Stethoscope, blood pressure, thermometer, pulsoximeter, balance - Paper-based folder containing the intervention manual; patient-reported outcome measures; key scientific articles related to the intervention components; list of contact information of regional cardio groups, physical activity groups, physiotherapists providing home therapies, home care services, mobile palliative care services
**Procedures**	**Describe each of the procedures, activities, and/or processes used in the intervention, including any enabling or support activities** Before discharge of hospitalization during recruitment into the study: Provision of the French or German version of the “Heart Failure Patient kit” brochure from the Swiss Heart Foundation (https://www.swissheart.ch); provision of patient self-care education Intervention during 90-day follow-up: 1. Nurse-patient direct contact a. Assessment of health status, self-care capabilities, and depressive symptomatology via patient self-completion of a series of questions using a web-based self-report tool, specifically developed for this study based on previous work, structured in four parts. To facilitate each instrument’s clinical applicability, we chose the shortest available version of each instrument i. One open question to assess a patient’s salient beliefs concerning living with HF (“What do you think of when you think of living with HF?”) ([35], p 6) ii. The 22-item Self-Care of Heart Failure Index (SCHFI) (French and German (for Switzerland) versions) to assess self-care capabilities [36] iii. The 12-item Kansas City Cardiomyopathy Questionnaire (KCCQ) (French and German versions) to assess disease-specific health status [37], plus a single item from the 23-item KCCQ to measure symptom stability [38]; and iv. The two-item Patient Health Questionnaire-2 (PHQ-2) (French and German versions) to assess depressive symptomatology [39] v. Nurse assessment of the patient’s main complaint and clinical assessment focusing on health status and fluid overload, including vital signs, pulmonary auscultation, peripheral oedema and the use of the V-scan hand-held ultrasound [40, 41] vi. Nurse-patient discussion of the assessments results 1. Review of the color-coded graphic results (generated of the web-based system according to the scoring and cut-off levels of the respective questionnaires), both on individual items and on each measured dimension (e.g. inadequate self-care maintenance; symptom frequency) 2. Evaluation of the clinical assessment results 3. In case of significant health deterioration [42] as judged by the nurse, immediate contact to the study cardiologist for medical evaluation 4. Nurse-patient exploration of priorities for support needs, according to the algorithm in the intervention manual considering assessment results and related risk for negative outcomes 5. Nurse provision of related counselling using a person-centred approach [43], teach-back techniques [44] and principles of motivational interviewing 6. Nurse suggestion of a follow-up procedure, ensuring appropriate care including a. Nurse contact and support to take appropriate actions and to make timely checks of the efficacy of the measure taken b. Encouragement of patient and family member to contact the nurse for guidance in interpreting symptoms 2. Write-up of the report on each relevant consultation, within one to seven days following the patient visit a. Review of assessment results, report and suggested follow-up procedures and provision of feedback on the results by the study’s Principal Investigator (PSK) to the intervention nurse 3. Discussion with the cardiologist following the patient visit a. Discussion between the intervention nurse and the study’s cardiologist (DG) of the nurse presented patient situation, follow-up procedures and report regarding the patient situation/main complaint, assessment results, interpretation, procedures and follow-up as well as in view of the medical plan and treatment where relevant 4. Provision of the collaborative written report to the general practitioner, the private cardiologist (if applicable), any home care personnel (if applicable), and the patient; upload to the hospital medical records archive
**Who provided**	**For each category of intervention provider (e.g. psychologist, nursing assistant), describe their expertise, background and any specific training given** 1. Provision of the brochure and patient self-care education during in-hospital phase: a. Provision of the brochure: Research assistants (all with a master-degree in nursing science) during recruitment b. Provision of patient self-care education: Registered nurses with a bachelor degree or equivalent with or without postgradual education in cardiology care, working in the cardiology clinic or at a medical ward at the hospital c. Specific preparation: Refresher on providing patient education principles for persons with HF including the appropriate use of the respective patient education materials (2-h team meeting) 2. Delivery of the intervention: a. Registered nurses with a bachelor or master degree, currently working as a nurse lecturer or scientific collaborator. Main professional clinical experience in internal medicine wards or intensive care unit of a teaching or university hospital. No previous experience in focused examination of the lungs using a hand-held pocket-sized ultrasound. Novice to the multicomponent intervention, but all with postgradual education in motivational interviewing, and experience in providing patient self-care education and conducting clinical assessment b. Specific preparation: i. Two-day education session on the intervention manual including a) the review and discussion of all intervention components; b) the use of the questionnaires for the evaluation of self-care capabilities, health status, and depressive symptomatology, the interpretation of results, scores as well as review of the algorithm for priority setting and activities; and c) the discussion of the delivery of the intervention using simulated scenarios; education provided by the principal investigator (PSK) who holds a PhD in Nursing Science and has professional experience as advanced nurse practitioner; assisted by a research assistant (JG) who holds a master degree in Nursing Science and has professional experience in palliative home-based care ii. One-day education session: refresher on clinical assessment, focus on the cardiovascular system; education provided by a nurse lecturer iii. One-day training on the correct use of the hand-held pocket-sized ultrasound device and interpretation of images of a focused lung examination for signs of fluid overload in view of detection of early decompensation; education provided by a physician of the emergency department of the hospital
**How**	**Describe the modes of delivery (e.g. face-to-face or by some other mechanism, such as internet or telephone) of the intervention and whether it was provided individually or in a group** - Face-to-face contact with a patient, accompanied or not by a family member - Telephone contacts via phone calls to the patient
**Where**	**Describe the type(s) of location(s) where the intervention occurred, including any necessary infrastructure or relevant features** - Clinic visits at the cardiology department of the hospital, in a separate room - Home visits at a patient’s home
**When and how much**	**Describe the number of times the intervention was delivered and over what period of time including the number of sessions, their schedule, and their duration, intensity or dose** During a period of 3 months, follow-up included 2.14 (mean, ± 0.97) visits per patient lasting a total of 166.96 min (mean, ± 72.55); and 3.1 (mean, ± 1.7) additional telephone contacts 0. Visit pre-intervention delivery before hospital discharge during recruitment period 1. First intervention delivery realized between day 7 and 15 after hospital discharge a. Schedule of a 1-h visit b. Normally scheduled at the cardiology outpatient; at the patient’s request, visit/s occurred at the patient’s home (e.g. for restricted mobility reasons) i. In cases of assessment of severe health problems that required immediate treatment (e.g. rapid worsening of dyspnoea), the nurse contacted to study cardiologist for medical evaluation 2. Further intervention delivery visits were scheduled on a needs-led basis during a period of 3 months (e.g. if further patient education was necessary, or in cases of unstable symptoms of worsening heart failure) 3. Additional telephone contacts were realized in case of following up activities taken against unstable symptoms a. At telephone contacts, in cases of severe health problems that required immediate treatment (e.g. rapid worsening of dyspnoea), patients were encouraged and, if necessary, assisted to contact their general practitioners and/or the emergency services
**Tailoring**	**If the intervention was planned to be personalized, titrated or adapted, then describe what, why, when, and how** - Consistent procedures and use of a person-centred approach [43] and motivational interviewing principles [30] - Personalized priority setting based on an individual’s assessment results of health status [37, 38, 42] and self-care capabilities [36]; and self-care support priorities on symptom stability [2], medication adherence [2], and physical activities [2] (see algorithms in the intervention manual). Provision of several follow-ups in the patient’s preferred setting, with tailoring of the intervention to fit individual situations, based on: - Objective and subjective information obtained via patient assessment - Each nurse’s clinical judgement and expertise concerning each patient’s situation and needs - Related follow-up priorities
**Modifications**	**If the intervention was modified during the course of the study, describe the changes (what, why, when, and how)** None
**How well**	**How well. Planned: If intervention adherence or fidelity was assessed, describe how and by whom, and if any strategies were used to maintain or improve fidelity, describe them** Assessment of intervention fidelity - Nurse report via completion of a 7-item paper-based checklist at the end of each relevant consultation Maintaining intervention fidelity - Review of the nurse documentation regarding each patient situation, main complaint, assessment results, intervention priorities, report of each consultation by the study PI; nurse-study PI discussion in case of disagreements - Nurse group discussions on patient situations and intervention priorities with the PI and a research assistant (JG) - Two group discussions with two HF nurse experts external to the core group
**Actual**	**Actual. If intervention adherence or fidelity was assessed, describe the extent to which the intervention was delivered as planned** Proportion of yes responses to the intervention components: (a) patient involvement in symptom monitoring and support for self-care capabilities; (b) facilitation of early decompensation detection; (c) optimized medical and device treatment following ESC guidelines; (d) psychosocial support for patient and family; (e) patient education; (f) easy access to care; and (g) facilitation of multidisciplinary collaboration.

Description according to the Template for Intervention Description and Replication (TIDiER) Checklist [45]

A core component of the intervention is supporting HF self-care practices which are hypothesized to activate cardioprotective mechanisms limiting inflammatory processes and reducing clinical congestion [46, 47]. This component includes the evaluation of each patient’s assessment data and any vulnerability characteristics relevant to self-care, in order to guide the provision of tailored support [23]. The intervention also includes the provision of a report summarizing health status and self-care assessment results and procedures, which is sent to all health care professionals providing usual care for the person. The intervention was delivered by nurses over a 3-month period. The first contact between intervention nurse and a person with HF occurred before hospital discharge. The first follow-up appointment was scheduled 7–15 days after hospital discharge. She then scheduled further visits on a needs-led basis (e.g. low self-care capability, poor health status or unstable symptoms), which took place in the cardiology outpatient setting, or at the person’s home for persons with restricted mobility.

To facilitate the consistent application of all intervention components, we placed emphasis on fidelity to recommended practices. At the same time, we encouraged nurses to tailor the intervention regarding frequency and duration of follow-up and setting, to fit people with HF’s individual needs and preferences and according to objective and subjective information obtained via patient assessment. The combination of fidelity to the intervention and tailoring according to patients’ needs and nurses’ expertise is inherent to complex nursing interventions [14, 18, 48] and is intended to ensure an effective, individualized intervention. Table 1 provides a summary of the intervention according to the Template for Intervention Description and Replication (TIDieR) Checklist [45]. The detailed description of the intervention components and process can be found in French and German in the relevant intervention manual (available on request from the first author)*.*

### Outcomes

#### Feasibility

The study’s feasibility was measured quantitatively using six criteria [27]:

1. Patient recruitment rate (percentage of eligible patients receiving study information and agreeing to participate)
2. Study nurse time needed for patient recruitment and inclusion in the study
3. Study attrition (percentage of participants who do not complete the patient-reported outcome (PRO) measures at 3-month follow‐up)
4. Fidelity to the intervention components assessed using a 7-item check‐list with dichotomous yes/no responses regarding each intervention component
5. The percentage of patients receiving one visit, additional telephone contacts and/or home visits and the percentage who received two or more such contacts
6. The mean duration of the average total patient visits and additional telephone contacts

#### Acceptability

Acceptability was assessed at 3-month follow-up in both groups via the 8-item Treatment Acceptability and Preference Questionnaire (TAPQ) [49], adapted for this study based on its French version [50] and the Sekhon et al. literature review and theoretical framework [51, 52] for multiple acceptability components (see Table 2). The 5-point response scale ranged from 0 (totally disagree) to 4 (totally agree).

**Table 2:** Acceptability results across five acceptability components at 3 months for participants in the intervention and control groups

Acceptability component	Item	Intervention (*n* = 18 to 19) Mean (SD)	Control (*n* = 15) Mean (SD)
**Affective attitude** How an individual feels about the intervention	These nursing consultations were appropriate in my situation	3.37 (0.96)	3.47 (0.52)
	I felt comfortable during these nursing consultations	3.58 (0.96)	3.40 (0.51)
	I was satisfied with these nursing consultations	3.53 (1.26)	3.40 (0.63)
**Burden** The perceived amount of effort that is required to participate in the intervention	I would participate again in these nursing consultations if the project was renewed	2.94 (1.26)	3.00 (1.51)
	Participation in these nursing consultations required a great deal of effort on my part	1.13 (1.54)	1.36 (1.15)
**Ethically** The extent to which the intervention has good fit with an individual’s value system	These nursing consultations were in line with my current values, which is important to me	3.37 (0.96)	3.40 (0.63)
**Opportunity costs** The extent to which benefits, profits or values must be given up to engage in the intervention	I had to give up some opportunities/occasions important to me to participate in these nursing consultations	1.36 (1.39)	1.20 (1.26)
**Perceived effectiveness** The extent to which the intervention is perceived as likely to achieve its purpose	These nursing consultations have been beneficial in helping me to live with my illness on a daily basis	3.44 (0.62)	3.47 (0.64)

Responses 0 = totally disagree 1 = disagree; 2 = neither disagree nor agree; 3 = agree; 4 = fully agree

#### Patient-reported outcomes

The intervention’s effect at 3-month follow-up was assessed using selected PRO and clinical outcomes. Although PRO measures do not always correlate with objective measures of biological or functional change, they are widely used as endpoints in clinical HF research, as they capture the patient’s perspective [53, 54].

##### Patient-reported outcomes

We measured *HF-specific self-care* via the French and German (for Switzerland) versions [25] of the 22-item Self-care of Heart Failure Index (SCHFI), v6.2 [33, 36, 55, 56], which measures self-care maintenance, self-care management and confidence over the past month. We standardized the scores for each subscale, and possible ranges were 0–100, with higher scores indicating better self-care, ≥ 70 suggesting adequate levels [36].

We measured *HF-related health status and symptom stability* over the past 2 weeks via the French and German versions of the 12-item Kansas City Cardiomyopathy Questionnaire (KCCQ) [37], with symptom stability measured via a single item from the 23-item version of the KCCQ [38, 57]. The KCCQ-12 contains a summary score (overall health status) and four domain scores (physical limitation, symptom frequency, quality of life, social limitation). We computed scores for a summary score as well as subscores for physical limitations, symptom frequency, quality of life and social limitations. We used one item from the KCCQ-23 version for symptom stability [37, 42]. We composed a clinical summary score [42] of physical limitation, and symptom frequency domain scores. Scores are scaled 0–100, where 0 is the lowest reportable health status and 100 the highest [37, 42]. To facilitate clinical interpretability and as recommended, we calculated the numbers/percentage of participants experiencing 5-, 10-, or 20-point changes from baseline to 3 months [42].

Finally, we measured *health-related quality of life* via the French and German versions of the 5-item EQ-5D-5L, including a VAS (Euroqol) [58]. The computed EQ-5D-5L scores include an overall score and subscores on five dimensions rating health on the day: mobility, self-care, usual activities, pain/discomfort and anxiety/depression, with scales ranging from 1 to 5, higher scores indicating higher severity/problems. EQ VAS provides a quantitative measure of the patient’s perception of overall health, with scores ranging from 0 (worst imaginable health) to 100 (best imaginable health) [58].

##### Clinical outcomes

The intervention’s effect on all-cause mortality, all-cause hospital admission and hospital length of stay was assessed for a 90-day period following initial hospital discharge as available from the hospital’s electronic medical records or GPs’ communications.

### Sample size

According to the CONSORT extension for pilot trials statement, no formal sample size calculation is required for pilot trials, but a rational should be given ([59], p. 4, 5). The minimum sample size for parametric statistical tests is often considered to be 30 per group [60], so we aimed for a total sample size of 60 persons with HF. We estimated this to be sufficient to evaluate feasibility (our study’s primary objective) and to calculate approximate effect sizes for a future large-scale trial.

### Stopping guidelines

We predefined criteria for the (premature) termination of the study as unresolvable severe and persistent failure to recruit patients into the trial, safety concerns, or alterations in accepted clinical practice that make the continuation of the study unwise. No other stopping rules or progression criteria were defined.

### Randomization; sequence generation

We used the Research Electronic Data Capture (REDCap) software’s randomization module for randomization (https://www.daunit.ch/en-us/). A scientific collaborator independent from the core research group (ALK) set up the web-based randomization process to assign eligible participants to intervention or control groups by remote allocation via REDCap. She first created the randomization table, generated sequences using ID and sample size parameters, and checked whether it had resulted in groups of similar sizes. Then, she uploaded the table as a locked up version for randomization into REDCap. No person directly involved in the study had access to allocation codes.

### Allocation concealment mechanism and implementation

The allocation sequence was concealed in REDCap. The principal investigator (PI) of the study (PSK) was the only person of the research group with allocation rights in REDCap and assigned patients after study consent and completion of baseline assessment. Group assignments were concealed and registered in REDCap within the respective participant ID.

### Recruitment and data collection

#### Recruitment

Research nurses screened daily lists of admitted patients for age and HF diagnosis and/or cardiac decompensation. They assessed further eligibility criteria via electronic medical records (DPI). If needed (e.g. in case the DPI notes included a cardiac pathology and typical HF symptoms but not HF diagnosis), they asked ward physicians or cardiologists to confirm or reject HF diagnosis. They collected data after inclusion in the study and before randomization at baseline and at 3-month follow-up. Safety was monitored for a further month after follow-up.

Study nurses obtained* socio-demographic and clinical variables* from the DPI, they completed forms for data not available in the DPI during a face-to-face interview and noted all data on a paper-based questionnaire. Then, participants completed baseline *paper-based outcome measures*; if participants preferred it, the research nurse entered their answers during a face-to-face meeting. All participants received basic patient information, i.e. the French or German version of “The Heart Failure Patient Kit” brochure by the Swiss Heart Foundation [29], from the research nurse. Then, the research nurses referred all participants to cardiology or ward nurses to receive patient education via one or two face-to-face encounters before discharge or up to 6 days post-discharge. Following randomization, the intervention nurse contacted intervention group participants during their hospitalization to establish contact and to schedule a follow-up appointment during the first or second week post-discharge. At 3-month follow-up, a research nurse sent paper-based questionnaires to participants. In case of missing returns, s/he reminded them with a fresh set of questionnaires, and phoned to inquire whether participants needed assistance for filling them in. Research nurses collected *clinical outcome data* from DPI and declarations by the participants’ GPs. Research nurses transcribed the data from paper-based forms and/or DPI into the REDCap data base which was double-checked for all outcome data.

### Blinding

Participants were not blinded towards group assignment, neither were intervention nurses, the study cardiologist or the PI of the study. Further, the PI informed health care professionals responsible for usual medical and nursing care about the participation of their patient without revealing group assignment, but sent consultation reports to health care providers of intervention group participants as well as uploaded them to hospital records. We did not inform cardiology or ward nurses who provided patient education of group assignment. Research nurses (MEV, GME) blinded towards group assignment managed the outcome data. Also, the statistician (KDH) conducting the analyses was not informed about group assignment.

### Statistical methods

#### Feasibility

To estimate the recruitment rate we calculated the percentage of eligible patients receiving study information and agreeing to participate. Research nurse time needed for patient recruitment and inclusion in the study was the sum of all time spent at this task (hours, minutes). The percentage of participants for whom we were unable to obtain PRO measures at 3-month follow-up determined the study retention/attrition rate. We expressed fidelity to the intervention components as an overall mean score of the percentage of “yes” responses to the fidelity checklist across all components and intervention delivery visits. We calculated the mean duration of patient visits, including time needed for preparation, direct contact, writing up the report and nurse-cardiologist discussions, as well as the mean duration of additional telephone contacts. We extended the recruitment period from an initially planned 35 weeks to roughly 94 weeks, to achieve the target sample, which was required for receiving funds from the external funders.

#### Patient-reported outcomes: heart failure self-care behaviour, disease-specific health status, health-related quality of life, all-cause mortality, all-cause admissions, and length of hospital stay

We calculated descriptive statistics for all variables, using proportions or measures of central tendency and dispersion as appropriate. We estimated effect sizes for the outcome variables: we calculated Cohen’s *d* for “self-care” (SCHFI V6.2), “HF-related health status” (KCCQ-12), “health-related quality of life” (Euroqol), “intervention acceptability” (adapted TAPQ), “length of stay” (LOS), and “number of readmissions” variables, and determined hazard ratios for “all-cause mortality” and “all-cause hospital admissions”. Kaplan–Meier analyses modelled the time to hospital admission and death. We applied intention-to-treat principles to the trial data. We did not impute missing values.

### Ethical considerations

For the present study, the local ethical commission considered any immediate risk to study participants as minimal (risk category A). We obtained ethical approval (CER-VD 2018–02156) and informed consent, and registered the study 5 months after enrolment of the first participant (study record: ISRCTN10151805). Also, we obtained ethical approval (CER-VD amendment 200609) on our request to continue the study during the COVID-19 period, including the uptake of recruitment after the Swiss lock-down period in spring 2020 with the use of appropriate safety protection measures, and the added exclusion criteria regarding SARS-CoV2 and COVID-19.

## Results

### Recruitment

**Fig. 1:**
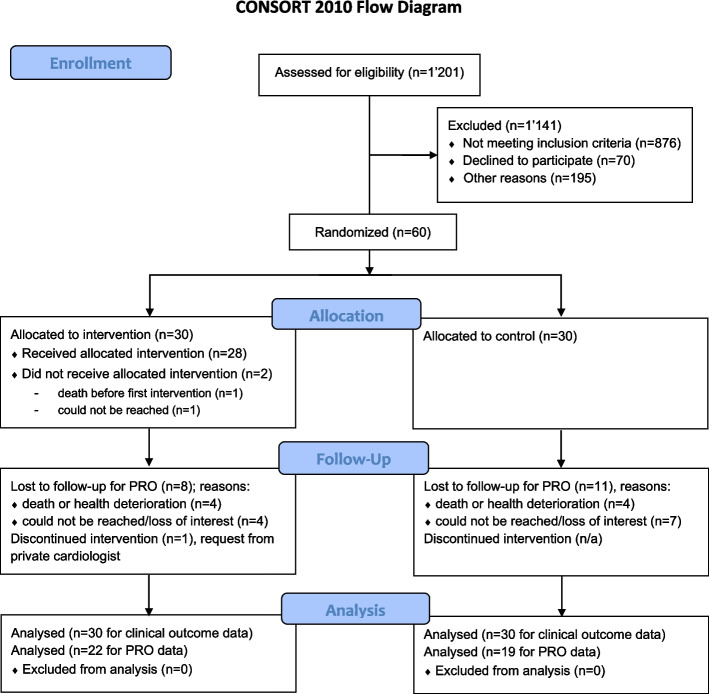
Participant flow with diagram. Recruitment at the main campus of the hospital occurred between 15.04.2019-04.02.2021; and at a second campus of the same hospital between 08.06.2020-04.02.2021. PRO=patient-reported outcomes. Consort-2010-Flow-Diagram: The EQUATOR Network (equator-network.org), access: 15.04.2022

Between 14 April 2019 and 4 February 2021, we screened hospital admission lists including 5314 patients admitted to the hospital, and excluded 4113 patients because of no HF diagnosis and/or age < 18 years. We then assessed the eligibility of 1201 patients, among which we enrolled 60 who provided informed consent. Of the remainder (*n* = 1141), 76.77% (*n* = 876) were ineligible, 6.14% declined to participate (*n* = 70) and 17.10% (*n* = 195) could not be enrolled for other reasons. Reasons for ineligibility (*n* = 876) were as follows: no hospitalization due to decompensated HF/no history of HF decompensation over the past 6 months (*n* = 557); ambulatory status (*n* = 174); cognitive impairment (*n* = 68); complicated serious comorbidity (*n* = 25); imminent life-threatening illness (*n* = 20); previous enrolment into the study (*n* = 18); not French or German speaking (*n* = 13); or family member of a research group member (*n* = 1). The most frequent reason for declining participation was lack of interest (*n* = 51), followed by fatigue (*n* = 10), and anxiety to participate in the study (*n* = 9). Other reasons for exclusion (*n* = 195) were as follows: early patient discharge (*n* = 56); discharge before confirmation of HF diagnosis was available to the study team (*n* = 47); discharge to a nursing home (*n* = 35); transfer to another facility (*n* = 28); no recruitment during end of year seasonal holidays (*n* = 16); death during hospitalization (*n* = 11); or other (*n* = 2) (Fig. 1). We recruited participants from eight internal medicine units of one campus and also from an internal medicine unit of a second campus of the same hospital. During Switzerland’s national lock-down period in spring 2020 related to the COVID-19 pandemic, no participant recruitment occurred between 12 March and 8 June 2020, and also during the following infection peak periods in autumn 2020.

### Baseline data

The sample consisted of 60 persons with HF (age mean = 75.7 years, SD = 8.9; 30% female; 63.3% in NYHA III-IV); Table 3 provides the sample’s demographic and clinical characteristics.

**Table 3:** Demographic and clinical characteristics at baseline of persons with heart failure in the intervention and control groups (*N* = 60)

	**Intervention (*n* = 30)** **Frequency (%) OR Mean (SD)**	**Control (*n* = 30)** **Frequency (%) OR Mean (SD)**
Age (in years)	75.67 (7.45)	75.75 (10.21)
Gender		
Men	20 (66.67)	22 (73.33)
Women	10 (33.33)	8 (26.67)
Nationality		
Swiss	28 (93.33)	27 (90.00)
Other European	1 (3.33)	2 (6.66)
Non-European	1 (3.33)	0 (0.00)
Education (*n* = 57)		
Obligatory school or less	6 (20.00)	7 (23.33)
Secondary school	14 (46.67)	10 (33.33)
Tertiary school	8 (26.67)	12 (40.00)
Enough resources to pay for medications and health care services (*n* = 58)		
Yes	26 (86.67)	26 (86.67)
No	3 (10.00)	3 (10.00)
Life situation (*n* = 59)		
Living alone	9 (30.00)	8 (26.67)
Living with someone	21 (70.00)	21 (70.00)
Support person (*n* = 59)		
Yes	29 (96.67)	28 (93.33)
No	1 (3.33)	1 (3.33)
Post myocardial infarction	9 (30.00)	14 (46.67)
Aetiology of heart failure		
Ischaemic heart disease	22 (73.33)	20 (66.67)
Hypertension	14 (46.67)	15 (50.00)
Valvular heart disease	12 (40.00)	11 (36.67)
Arrythmia	17 (56.67)	16 (53.33)
Cardiomyopathy	2 (6.67)	3 (10.00)
Other	2 (6.67)	2 (6.67)
Comorbidity		
Diabetes	10 (33.33)	10 (33.33)
Hypertension	19 (63.33)	16 (53.33)
Cerebrovascular disease	3 (10.00)	9 (30.00)
Renal disease	12 (40.00)	21 (70.00)
Cancer	8 (26.67)	2 (6.67)
Depression/anxiety	5 (16.67)	9 (30.00)
Cognitive impairment	1 (3.33)	1 (3.33)
Charlson Comorbidity Index (%)	5.97 (1.96)	6.86 (1.75)
NYHA (*n* = 59)^a^		
II	13 (43.33)	8 (26.67)
III	11 (36.67)	13 (43.33)
IV	6 (20.00)	8 (26.67)
Left ventricular ejection fraction (EF) (%)	38.89 (15.59)	36.06 (14.59)
HFpEF^a^	17 (60.71)	20 (66.67)
HFmrEF^a^	2 (7.14)	4 (13.33)
HFrEF^a^	9 (32.14)	6 (20.00)
Systolic BP (mmHg)^a^	119.50 (17.61)	115.87 (14.34)
Treatment		
Angiotensin-converting enzyme inhibitor (ACE-I)	10 (33.33)	6 (20.00)
Angiotensin II receptor blockers (ARB)	7 (23.33)	8 (26.66)
Beta-blocker	27 (90.00)	24 (80.00)
Diuretic	27 (90.00)	24 (80.00)
Digoxin/digitalis	1 (3.33)	0 (0.00)
Mineralocorticoid receptor antagonist (MRA)	9 (30.00)	10 (33.33)
Angiotensin receptor-neprilysin inhibitor (ARNI) (Entresto)	6 (20.00)	6 (20.00)
Anticoagulant	13 (43.33)	16 (53.33)
Calcium channel blocker (CCB)	5 (16.66)	6 (20.00)
Antihypertensive agents, vasodilators (Minoxidil)	0 (0.00)	1 (3.33)
Vasodilators (nitrates)	1 (3.33)	3 (10.00)
Coronary vasodilator	0 (0.00)	1 (3.33)
Antiarrhythmic (amiodarone)	1 (3.33)	3 (10.00)
Thiazide diuretic with potassium-sparing diuretic (comilorid)	1 (3.33)	0 (0.00)
Sleep disturbance		
Yes	17 (56.67)	14 (46.67)
No	13 (43.33)	16 (53.33)
Daytime sleepiness		
Yes	6 (20.00)	10 (34.48)
No	24 (80.00)	19 (65.52)
LOS index hospitalization (days)	17.83 (18.63)	11.17 (7.38)

^a^ *NYHA* New York Heart Association Classification, HF with preserved ejection fraction, HFpEF (≥ 50% EF); HF with mildly reduced EF, HFmrEF (41–49% EF); HF with reduced ejection fraction, HFrEF (≤ 40% EF), *BP* Blood pressure, *LOS* Length of hospital stay

### Numbers analysed

Outcome data were analysed for 60 persons with HF regarding clinical outcomes; PROs at 3-month follow-up were available and analysed for 22 IG and 19 CG participants (Fig. 1).

### Feasibility

1. *Patient recruitment rate* was 46.15%. Of 130 eligible patients receiving study information, 70 declined study participation and 60 agreed to participate and provided written consent. Patients declared reasons for declining were no interest if there was a 50% chance of being in the control group (*n* = 51, 72.9%); fatigue (*n* = 10, 14.3%); and anxiety in view of study participation (*n* = 9, 12.8%).
2. *Study nurse time needed for patient recruitment and inclusion in the study.* Over the 62-week recruitment phase, compounded by the ongoing COVID-19 epidemic, research nurse time totalled 1011.4 h for patient recruitment, including 380.75 h for screening and eligibility assessment, 105.15 h for providing study information and 525.5 h for obtaining informed consent for study participation.
3. *Study attrition.* Study attrition rate was 31.7%. There was no patient withdrawal from the study. However, after delivery of the first intervention with the communication of the intervention report, a private cardiologist requested to withdraw his patient from the intervention. We were able to obtain clinical outcome data from all 60 participants, but PRO data only from 41 of them (PRO data was missing from 11 CG and 8 IG participants, Fig. 1). Therefore, the study attrition rate was zero for clinical outcome and 31.7% for PRO data.
4. *Fidelity to the intervention components.* Nurses’ reported fidelity to the intervention components was 0.71 (± 0.05). Mean fidelity to all intervention components at the first, second, third, fourth, and fifth intervention delivery was 0.70 (± 0.12), 0.71 (± 0.11), 0.68 (± 0.10), 0.71 (± 0.11), and 0.74 (± .), respectively. Across all intervention delivery visits, highest mean fidelity was reported for facilitation of early decompensation detection (0.89, ± 0.22), followed by patient education (0.85, ± 0.23) and patient involvement in symptom monitoring & self-care capabilities support (0.84, ± 0.16), and lowest mean fidelity was reported for multidisciplinary collaboration facilitation (0.46, ± 0.23) (Fig. 2, Table 4).

**Fig. 2:**
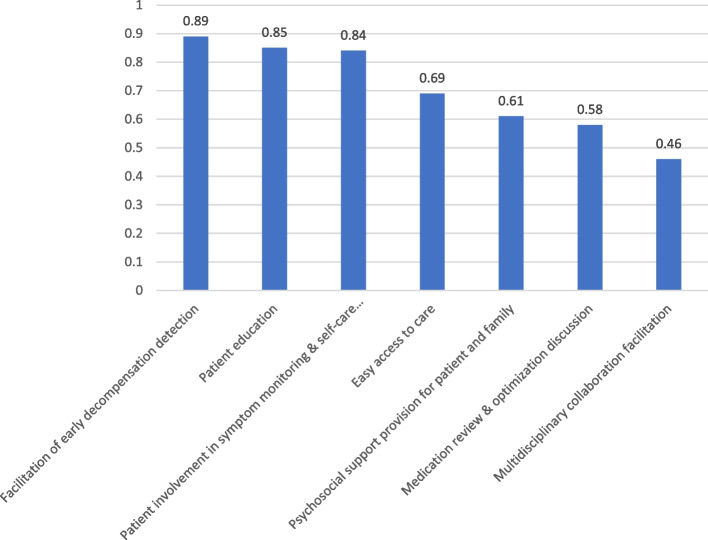
Intervention fidelity across all visits for the intervention components (proportion of yes responses)
5. *The percentage of patients receiving one visit, additional telephone contacts and/or home visits and the percentage who received two or more such contacts.* Follow-up included a mean of 2.14 (± 0.97) visits per patient (clinic visits 1.2 (mean, ± 1.2) and 1.9 (mean, ± 1.2) home visits) and a mean of 3.1 (± 1.7) additional telephone contacts. Of the 30 persons with HF intervention group sample, 70% (*n* = 21) received more than 1 visit, 23.33% (*n* = 7) received one visit, and 6.66% (*n* = 2) received none. Of 28 participants who received at least one visit, 64.29% (*n* = 18) received home visits only, 25% (*n* = 7) clinic visits only, and 10.71% (*n* = 3) both. Also, two participants received the initial face-to-face visit first, then telephone calls only during the remaining time of their follow-up period due to the COVID-19 lock-down period.
6. *The mean duration of patient visits and additional telephone contacts.* The follow-up period lasted from April 2019 to May 2021. The total time spent by a nurse for a patient over all visits was on average 166.96 min (± 72.55) including preparation, direct contact, report, and coordination with usual care. Single visits lasted a mean 80.25 (± 14.53) min. A telephone contact lasted on average 21.3 min (± 14.6). The total mean time of telephone contacts amounted to 31.2 (± 52.55) min per patient. The total duration of clinic visits, home visits plus additional telephone contacts together was 215.1 min (± 75.5) per patient on average over the 3-month follow-up period.

**Table 4:** Fidelity to the intervention components (proportion of yes responses)

Intervention component	Intervention delivery visits^a^			
	**1** ***n*** ** = 28**	**2** ***n*** ** = 23**	**3** ***n*** ** = 15**	**4** ***n*** ** = 5**
Patient involvement in symptom monitoring & self-care capability support provision	0.84 ± 0.17	0.84 ± 0.16	0.85 ± 0.17	0.83 ± 0.12
Facilitation of early decompensation detection	0.87 ± 0.23	0.91 ± 0.65	0.87 ± 0.25	1.00 ± 0.00
Medication review & optimization discussion	0.54 ± 0.27	0.65 ± 0.28	0.53 ± 0.23	0.70 ± 0.27
Patient education	0.87 ± 0.21	0.80 ± 0.30	0.87 ± 0.19	0.95 ± 0.11
Psychosocial support provision for patient and family	0.62 ± 0.28	0.57 ± 0.32	0.67 ± 0.50	0.47 ± 0.30
Easy access to care	0.75 ± 0.31	0.76 ± 0.31	0.50 ± 0.45	0.53 ± 0.38
Multidisciplinary collaboration facilitation	0.44 ± 0.20	0.46 ± 0.27	0.47 ± 0.20	0.47 ± 0.30

^a^ There was a total of 72 fidelity checklists provided across 5 visits. For the 5th visit, there was one checklist, with means of 1.00 for all intervention components except multidisciplinary collaboration facilitation (mean = 0.33), medication review & optimization discussion (mean = 0.50), and psychosocial support provision for patient and family (mean = 0.67) and easy access to care (mean = 0.67)

### Acceptability

Acceptability summary scores were high across all components, in both groups. Highest scores in the intervention group were for items linked to affective attitude (e.g. I felt comfortable, mean = 3.58). Regarding the burden component, participation in the intervention visits were not perceived as requiring a great effort; but the intention to participate again in the project if it should be reconducted received the lowest score (mean = 2.94). The control group provided similar ratings (Table 2). There was no effect of the intervention on acceptability (summary score, Cohen’s *d* =  − 0.071).

### Heart failure self-care behaviour, HF-related health status, health-related quality of life, all-cause mortality, all-cause admissions, and length of hospital stay

At baseline, one study participant did not provide responses to the PRO questionnaires due to fatigue; at 3 months, 19 study participants (IG = 8, CG = 11) did not provide responses to the PRO questionnaires. Reasons for missing PRO questionnaires at 3 months were as follows: health deterioration or death (*n* = 8), loss of interest in responding to questionnaires (*n* = 3), and non-response/unknown reasons (*n* = 8).

Table 5 presents descriptive results for HF-specific self-care, HF-related health status including symptom stability, and health-related quality of life.

**Table 5:** Descriptive results of self-care, HF-related health status, health-related quality of life at baseline and after 3 months of patients with heart failure in the intervention and control groups (*N* = 60)

	**Time**	**Intervention (*n* = 30)** **Mean (SD)**	**Control (*n* = 30)** **Mean (SD)**
Self-care (SCHFI v6.2)^a^			
Self-care maintenance subscale score	BL	59.06 (16.50)	59.43 (18.30)
	FU	69.58 (19.76)	67.38 (19.68)
Self-care management subscale score	BL	32.58 (26.92)	37.04 (35.05)
	FU	70.98 (32.02)	53.99 (31.68)
Self-care confidence subscale score	BL	78.27 (20.49)	76.29 (18.87)
	FU	78.96 (22.23)	86.40 (16.19)
Health status (KCCQ-12)^a^			
Physical limitation score	BL	52.62 (22.74)	54.91 (24.51)
	FU	63.33 (22.52)	61.18 (26.93)
Symptom frequency score	BL	49.15 (21.27)	40.09 (25.20)
	FU	69.06 (25.64)	72.59 (27.41)
Quality of life score	BL	42.13 (28.41)	48.28 (25.60)
	FU	55.63 (29.93)	59.87 (30.78)
Social Limitation score	BL	56.79 (34.61)	55.06 (34.12)
	FU	67.50 (27.82)	59.43 (26.42)
Summary score	BL	50.17 (20.76)	49.25 (22.19)
	FU	63.88 (23.11)	63.27 (23.58)
Clinical summary score	BL	50.89 (17.96)	47.36 (21.71)
	FU	66.20 (21.38)	66.89 (23.05)
Symptom stability item (item from KCCQ-23)	BL	46.15 (32.92)	43.10 (34.65)
	FU	60.00 (18.47)	68.75 (33.92)
Health-related quality of life (EQ-5D-5L)^a^			
Mobility	BL	2.12 (1.11)	2.45 (1.24)
	FU	2.25 (1.02)	2.84 (1.01)
Self-care	BL	1.26 (0.71)	1.52 (0.91)
	FU	1.15 (0.37)	1.47 (0.70)
Usual activities	BL	2.04 (1.04)	2.38 (1.12)
	FU	1.95 (0.83)	2.32 (0.89)
Pain/discomfort	BL	1.84 (1.90)	1.97 (0.98)
	FU	1.90 (0.91)	2.05 (0.97)
Anxiety/depression	BL	1.81 (0.92)	1.72 (0.88)
	FU	1.80 (0.95)	1.84 (1.34)
Perception of overall health (EQ VAS)	BL	61.70 (16.34)	58.03 (16.13)
	FU	66.74 (20.76)	67.22 (21.02)

The average difference between BL and FU (if drawn from the means depicted in this table) is not the same as the difference of the averages at those times (reported in the text). This can be attributed to missing data at FU

*BL* Baseline, *FU* Follow-up

^a^ Higher SCHFI subscale scores mean higher self-care with ≥ 70 cut-off for adequate self-care levels; higher KCCQ-12 domain and summary scores mean higher reportable health status; higher EQ-5D-5L scores indicate higher severity/problems, higher EQ VAS scores mean higher imaginable health

#### Self-care results for the SCHFI V6.2 (self-care maintenance, self-care management, and self-care confidence)

**Fig. 3:**
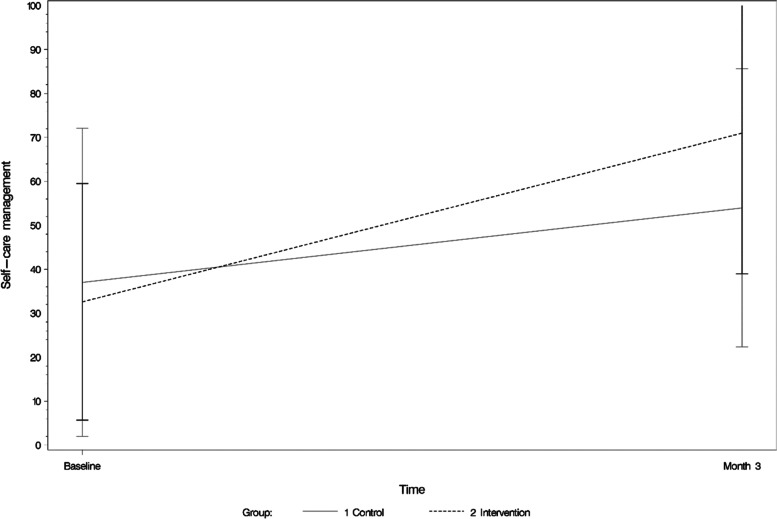
Self-care management (as measured via SCHFIv6.2) in the intervention and control groups

At baseline, participants in both the intervention and control groups showed inadequate levels (< 70 points) for self-care maintenance and self-care management; with similar self-care maintenance levels in both groups but lower self-care management levels in the intervention group compared to the control group. After 3 months, the participants in the intervention group had improved their *self-care maintenance* levels by 13 points compared to 8 points for the control group, thus approaching adequate self-care maintenance levels (IG = 69.6; CG = 67.4). At 3 months, *self-care management* levels were 71 for the IG and 54 for the CG, after an increase of 36.1 points in the intervention group compared to 16 points in the control group (Fig. 3). In contrast, self-care confidence levels at baseline were adequate in both groups (IG = 78.3, CG = 76.3), at 3 months they had increased by 2.7 points and 10.1 points in the intervention and control groups, respectively (IG = 79; CG = 86.4).

There were positive effect sizes for self-care maintenance (Cohen’s *d* = 0.216) and self-care management (Cohen’s *d* = 0.594) in favour of the intervention group and a negative effect size for self-care confidence (Cohen’s *d* =  − 0.387) (Table 6).

**Table 6:** Intervention effect sizes of patient-reported outcomes, hospitalizations and length of stay

Variable	2 groups, *n*	S^2^(*n* − 1)	Pooled std	Cohen’s *d*
**Self-care (SCHFI v6.2)**				
Self-care maintenance	37	18,510.26	23.00	**0.216**^**a**^
Self-care management	23	23,878.32	33.72	**0.594**^**b**^
Self-care confidence	37	20,836.67	24.40	** − 0.387**^**c**^
**HF-related health status (KCCQ-12)**				
Health status, overall score	37	21,627.70	24.86	**0.195**^**a**^
Physical limitation score	36	26,892.20	28.12	**0.363**^**a**^
Symptom frequency score	37	33,290.14	30.84	** − 0.206**^**c**^
Quality of life score	37	25,518.00	27.00	**0.318**^**a**^
Social limitation score	36	45,628.71	36.63	**0.232**^**a**^
Symptom stability item^e^	25	56,962.91	49.77	** − 0.160**
Clinical summary score^f^	36	21,987.12	25.43	**0.078**
**Overall self-rated health status for the EQ VAS**	35	16,750.62	22.53	** − 0.107**
**Number of hospitalizations**	60	31.21	0.73	** − 0.222**^**ad**^
**Length of hospital stay (LOS)**	60	7,056,550	348.80	** − 0.325**^**ad**^

Positive effect at a small^a^ or medium^b^ size; ^c^negative small effect size; ^d^a negative effect size for hospitalization and the LOS variable favours the intervention group; ^e^a single item from the 23-item version of the KCCQ; ^f^calculated as following: (physical limitation score + symptom frequency score)/2

#### Health status results for the KCCQ-12

Overall health status improved from baseline to follow-up by 18.9 (mean, ± 21,5) in the intervention compared to 14.10 (mean, ± 28.0) in the control group. Regarding domain scores, the improvements for the physical, social, symptom frequency, quality of life, clinical summary, and symptom stability scores were respectively 15.4 (mean, ± 26.7), 26.9 (mean, ± 29.3), 20.4 (mean, ± 21.7), 13.2 (mean, ± 34.1), 21.11 (mean, ± 22.04), and 12.50 (mean, ± 46.77) for the intervention group and were 5.2 (mean, ± 21.8), 33.2 (mean, ± 32.4), 11.8 (mean, ± 31.6), 4.7 (mean, ± 39.3), 19.12 (mean, ± 28.77), and 20.45 (mean, ± 53.41) for the control group. All effect sizes were small with largest sizes for the physical limitation score (Cohen’s *d* = 0.36) and QoL score (Cohens’ *d* = 0.32), and were in favour of the IG except for symptom frequency and symptom stability (Table 6).

**Fig. 4:**
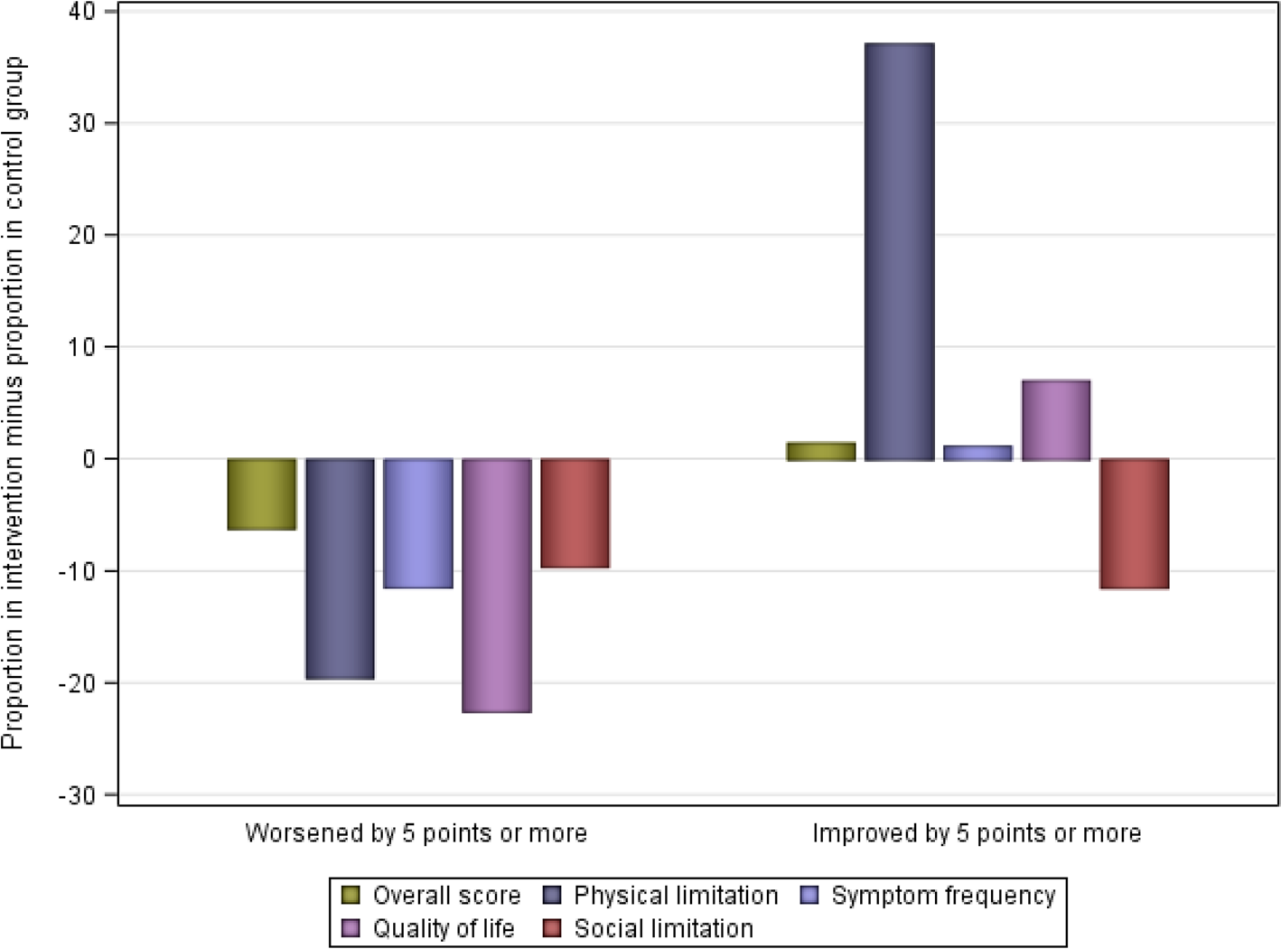
Difference in HF-related health status between study groups

The distribution of participants experiencing 5-, 10-, or 20-point changes, which are of clinical relevance [42], are presented in Table 7. There were more participants whose KCCQ scores over time did not worsen by 5 points or more; or improved by 5 points or more in the intervention compared to the control group across all scores, except for an improvement in the social limitation score (Fig. 4).

**Table 7:** Distribution of proportion of the KCCQ overall score and domains scores in the intervention and control groups

KCCQ	Change between BL and FU	Intervention group *n* = 19		Control group *n* = 17–18	
		**%**	***n***	**%**	***n***
Health status, overall score	Worsened by ≥ 5 points	10.52	2	16.67	3
	Did not change	15.79	3	11.11	2
	Improved by ≥ 5 points	73.68	14	72.22	13
Physical limitation score	Worsened by ≥ 5 points	15.79	3	35.25	6
	Did not change	0	0	17.65	3
	Improved by ≥ 5 points	84.21	16	47.05	8
Symptom frequency score	Worsened by ≥ 5 points	5.26	1	16.68	3
	Did not change	15.79	3	5.56	1
	Improved by ≥ 5 points	78.95	15	77.78	14
Quality of life score	Worsened by ≥ 5 points	5.26	1	27.78	5
	Did not change	21.05	4	5.56	1
	Improved by ≥ 5 points	73.69	14	66.66	12
Social limitation score	Worsened by ≥ 5 points	31.59	6	41.17	7
	Did not change	21.05	4	0	0
	Improved by ≥ 5 points	47.37	9	58.82	10
Symptom stability item (IG, *n* = 14; CG, *n* = 11)^a^	Worsened by ≥ 5 points	28.57	4	36.36	4
	Did not change	14.29	2	18.18	2
	Improved by ≥ 5 points	57.14	8	45.45	5
Clinical summary score^b^	Worsened by ≥ 5 points	5.26	1	11.76	2
	Did not change	21.05	4	11.76	2
	Improved by ≥ 5 points	73.69	14	76.46	13

^a^ A single item from the 23-item version of the KCCQ

^b^ Calculated as following: (physical limitation score + symptom frequency score)/2

#### Health-related quality of life

Participants’ perceptions of overall health for the EQ VAS was 61.70 (mean, ± 16.34) and 58.03 (mean, ± 16.13) in the intervention and control groups at baseline and 66.74 (mean ± 20.76) and 67.22 (mean, ± 21.02) in the intervention and control groups after 3-month FU. Health-related quality of life for the EQ-5D-5L overall score was 1.81 (± 0.68) and 2.01 (mean, ± 0.66) at baseline in the intervention and control groups and 1.81 (mean ± 0.63) and 2.11 (mean, ± 0.67) at 3-month FU in the intervention and control groups. The effect size was small for health-related quality of life (Table 6).

#### All-cause mortality, all-cause hospital admission, and hospital length of stay

Three months after inclusion, five persons with HF had died (IG = 3, CG = 2). There were 13 hospital admissions in the intervention group and 18 in the control group over the 3-month follow-up period. The intervention and control groups spent on average 8.90 days (median, IQR = 9.70) and 15.38 days (median, IQR = 18.41) per patient, respectively, in hospital (Table 8). The effect size for admissions was − 0.22 (Cohens’ *d*), and for days spent in hospital − 0.33 (Cohens’ *d*), meaning that the intervention had a small positive effect leading to fewer admissions and a shorter length of stay (Table 6).

**Table 8:** Clinical events in the intervention and control groups after 90 days follow-up

Clinical events	Intervention (*n* = 30) Frequency OR median (IQR)	Control (*n* = 30) Frequency OR median (IQR)
Number of deaths	3	2
Number of all-cause admissions	13	18
Time to death (days)	69.00 (43.00)	18.50 (7.00)
Time to first hospitalization (days)	20.00 (44.00)	28.50 (56.00)
Time (days) in hospital per patient across all hospitalizations	8.90 (9.70)	15.38 (18.41)

**Fig. 5:**
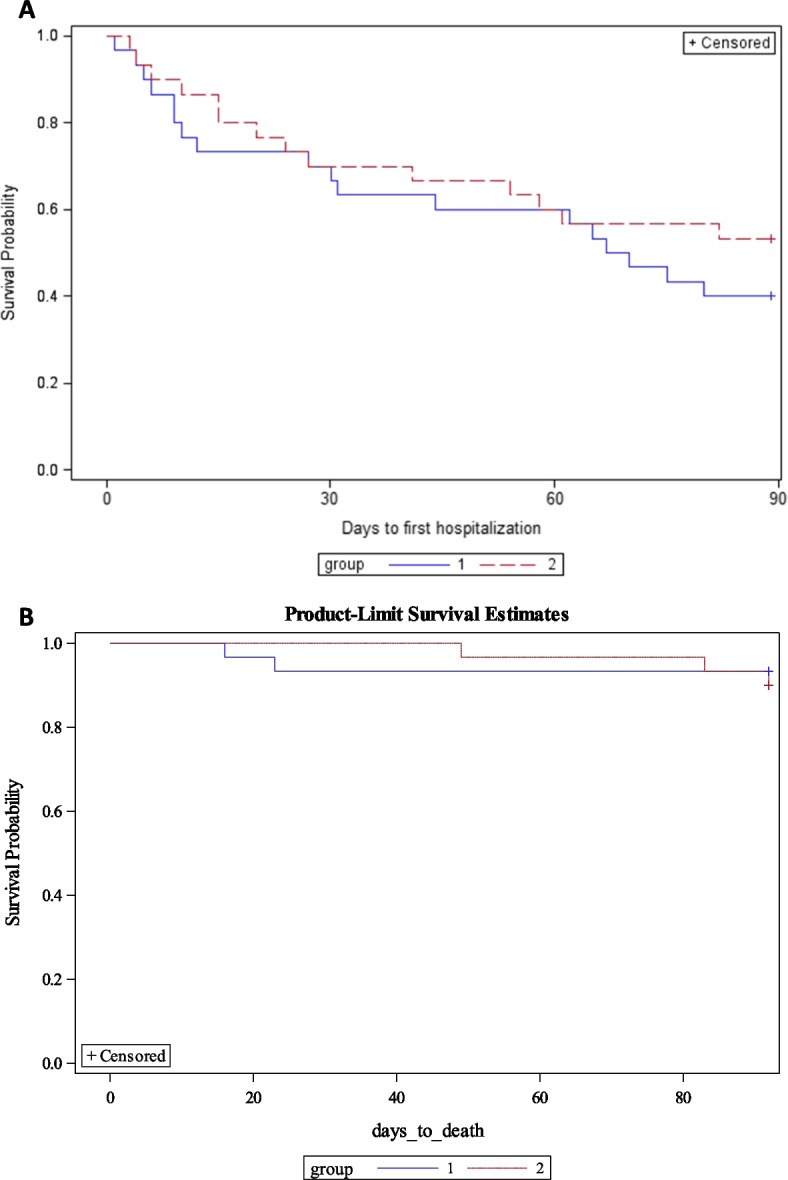
**a** Kaplan–Meier curve for days to first hospitalization for the control (group 1) and intervention group (group 2). **b** Kaplan–Meier curve for all-cause mortality for the control (group 1) and intervention groups (group 2)

Time to death was 69.00 (median, IQR = 43) days in the intervention group and 18.50 (median, IQR = 7) days in the control group. Time to first admission was 20.00 (median, IQR = 44) days in the intervention group and 28.50 (median, IQR = 56.00) days in the control group. The risk of all-cause admission was lower in the intervention group than in the control group (HR = 0.72; 95% CI = 0.43–1.45) (Fig. 5a). A hazard ratio estimate of all-cause mortality was not possible because of the low number of observed fatalities. The Kaplan–Meier curve for the low number of deaths (Fig. 5b) is suggestive of deaths occurring later in the intervention group than the control group. However, due to a death occurring at the end of follow-up (day 89), the total number of deaths was higher in the intervention group than in the control group (Table 8).

### Required sample size

Based on this study’s findings, a mono- or multicentre trial would require respectively 304 or 751 participants (across ten centres) for HF-related QoL (effect size = 0.3) at an alpha level of 0.05 and a power of 0.80.

### Ancillary analyses

None performed.

### Harms

No serious adverse events related to the intervention or study procedures were observed during the 4-month monitoring period. The study procedures and the intervention therefore appear to be safe.

## Discussion

This pilot RCT was intended to test the feasibility of a novel multicomponent complex intervention for supportive follow-up of persons with HF in Switzerland. Another goal was to provide information on patient-reported and clinical outcomes to inform the design of a subsequent fully powered RCT to evaluate the effectiveness of the intervention. The results indicate high feasibility and acceptability and provide estimates of effect sizes (Cohen’s *d*), which is rare in this field. The study’s recruitment rate (46%) and recruitment progress (slow) indicate that either a large single centre or multiple centres will be required for the conduction of a subsequent large-scale trial. Our study’s strict inclusion criteria targeted a very specific group of HF persons, namely those with current or recent decompensation. Our intervention targets these persons. Unexpectedly, we experienced difficulties in obtaining HF diagnoses sufficiently quickly to enable recruitment of the participants. While many factors may contribute to the availability of HF diagnosis [61, 62], about which a detailed discussion would be beyond the scope of this paper, effective recruitment depends on it. This confirms the importance of conducting pilot feasibility studies “to test the waters” ([14], p 166) in order to prevent the failure of large trials due to problems such as recruitment issues.

### Feasibility

The study’s recruitment period spanned roughly 2 years with a 62-week period of effective recruitment to reach the target sample size of 60 persons with HF, a duration which we had underestimated based on our previous study where we recruited 310 persons with HF over a similar time period [25]. First, the COVID-19 pandemic began during the study’s recruitment period and was followed by severely restricted access to the study sites during lock-down in spring 2020 and subsequent infection peak periods. However, the recruitment rate was similar before the onset of the COVID-19 pandemic and after the related lock-down or infection peaks; therefore, we believe that the pandemic had no influence on the recruitment rate. Second, the high number of patients excluded for reasons other than eligibility criteria included 84 patients who were discharged or transferred to another facility before receiving study information, and 47 patients who were discharged before HF diagnosis confirmation was available to our study team. These findings are indicative of a mismatch between study nurses’ working hours, time of physicians’ communication of HF diagnosis confirmation, and patient hospital discharge. Additionally, providing study information, responding to questions, and obtaining consent at a convenient time for the hospitalized person and for the care team was a highly time-consuming process. Involving research nurses who can allocate a higher percentage of their working time to the study would be useful, especially in settings with rapid discharge decisions. Further, sufficient capacity for HF diagnosis is crucial for effective recruitment. A greater involvement of cardiologists in the recruitment process would be indicated. Third, the percentage of non-included patients assessed for eligibility was higher in this study compared to our previous study (95% vs. 65%), which had a cross-sectional design [25]. A direct comparison of our results to other studies is difficult due to variations in the target sample, recruitment in specialized or general wards, reported numbers, and differences in the nature of the intervention itself. Nevertheless, our study’s recruitment rate and duration were similar to another study conducted in a Swiss University hospital, where Leventhal et al. [11] reported the inclusion of 42 participants (out of 140 eligible patients) over a 20-month period, signalling a 30% inclusion rate. In contrast, Strömberg et al. [63] reported a 66% inclusion rate (106 participants out of 161 eligible patients), while Dracup et al. [19] recruited 614 participants within 50 months, thus reporting a 96.5% inclusion rate. The low recruitment rate reported in the two Swiss studies, although 10 years apart, draws our attention towards carefully addressing recruitment issues ahead of time, to prevent the failure of large-scale trials due to insufficient participation [14]. The issues around recruitment may be also linked to recruitment in general wards, underdiagnosis [61, 62, 64, 65], and/or the absence of any HF registry in Switzerland.

We observed a considerable attrition rate regarding filling-in PRO measures at 3 months follow-up, particularly in the control group. Missing PRO measures is a common problem across clinical studies and a variety of minimizing strategies have been proposed [66]. They include an appropriate oversampling in future studies, to account for non-returned PRO measures. Another possibility would be to tailor data collection procedures for PROs according to participants’ preferences. For example, filling-in PRO measures could occur during regular follow-up appointments, accompanied by a reminder call, or with assistance from a research nurse. Patient and Public involvement (PPI) has shown to positively impact enrolment and retention in clinical trials [67]. In future studies, we believe that PPI [67–69] regarding recruitment, study procedures, and materials will be key to success.

The proportion of positive responses on the fidelity checklist was similar/consistent across visits. However, it varied between intervention components. This suggests suboptimal fidelity to the intervention as a whole, with some components and active ingredients most probably underdelivered. Many factors affecting fidelity exist at individual, local, and national levels [70, 71]. They include consistency of the intervention with current practice, availability of resources, leadership, training, or effective monitoring [70, 71], all of which may have been influential in this study. Intervention fidelity has been recognized as essential to increase scientific confidence that a change in target outcomes is attributable to the intervention under investigation [72]. A range of intervention fidelity components and strategies have been recommended [72]; some related to “training the providers” and “delivery of the intervention” [72] were used in this study. Enhancing and/or intensifying intervention fidelity strategies are likely to be valuable in increasing fidelity. Finally, we assessed fidelity to the implementation of the intervention with regard to adherence to the content of the intervention and frequency of delivery, and via self-report by nurses. Measuring all subcategories of adherence including content, frequency, duration, and coverage/dose [73] as well as including observations and researchers’ monitoring [74] should provide a more complete view on implementation fidelity.

We further explored the number of visits and duration of intervention delivery and found that the majority of participants received more than one visit, and more visits at home than at the clinic. Except for the first visit, defined in the protocol as taking place within 7 to 15 days post hospital discharge [1], nurses scheduled visits on a needs-led basis. In line with the 2021 ESC guidelines [1], these findings suggest that persons with HF need ongoing and regular supportive follow-up in line with self-care capabilities and symptom stability. The majority of visits were delivered at home, which was unexpected. Home visits by nurses have previously shown to be effective in HF follow-up care [5, 75] and as being the preferred way of engagement for most persons with HF [76]. The provision of both home and clinic visits by same intervention nurses may be a challenge in usual care. Stakeholder involvement to discuss and address related barriers and facilitators for conducting home visits would therefore be useful for future studies [18].

The time required for delivering a single session was on average 1.5 h. This time includes direct contact delivering the intervention as well as preparation (reviewing the patient’s medical and intervention notes), writing up the report, nurse-cardiologist discussions, and coordination with usual care. We consider all these parts as necessary. We are aware that this far exceeds the time available in usual care [77]; however, we could not identify any time-saving opportunities. The intervention’s cost-effectiveness should also be evaluated, which was beyond the scope of this study but might be part of a subsequent study [18].

### Acceptability

The results indicate generally high acceptability towards the intervention and study participation. The lowest acceptability ratings were for perceived burden related to study participation. This finding indicates that the real and perceived burdens of participating in the study need to be addressed. This burden includes filling-in PRO measures as repeated measurements. As we already used short versions of validated questionnaires in this study, PPI may help to identify additional options for decreasing study participation burden.

### Patient-reported and clinical outcomes

We estimated that the intervention had small to medium effects on self-care, HF-related health status, perception of overall health, admissions, and length of stay. These results suggest a positive outcome responsiveness for the intervention, whose priorities were early decompensation detection and self-care capability support such as managing symptoms and maintaining and increasing physical activities. However, these findings only provide approximations for the future performance of the intervention, given this pilot study’s small sample size and wide confidence intervals.

Nevertheless, the study’s effect sizes were clinically relevant for target outcome variables. More specifically, for self-care, a difference of 8 points has been defined as clinically relevant [36] and adequate self-care management with improved symptom response have been related to event-free survival [78] or fewer clinical events [79]. In this study, we found a 20-point difference between the intervention and control groups from baseline to follow-up with the intervention group reaching an adequate level of self-care management. Further, the intervention seems to be promising for HF-related physical limitation and quality of life. Physical activity and exercise are recommended self-care activities [1] and improving quality of life is among the three major treatment goals for persons with HF with reduced left ventricular ejection fraction [1]. Additionally, our examination of proportions of participants with clinically important changes in health status over time [42] showed that fewer intervention group participants saw their HF-related health status worsen compared to the control group. Importantly, not worsening HF is a central objective of HF care [1] including recognizing it [80] as most persons with HF have episodes of worsening of HF [81]. Also, in view of treatment goals, “not getting worse” has been named to matter most for patients, including improving physical function, decreasing symptoms, avoiding readmissions, and being able to live a normal life [82].

There were three deaths in the intervention group and two deaths in the control group, which may raise concerns. At each visit, the intervention included health status assessment via clinical assessment, the KCCQ (whose scores are interpretable and strongly associated with clinical events) [42, 83], and information on pulmonary congestion through the use of a pocket-sized ultrasound device [40, 41] (Table 1). The intervention nurse communicated any findings to the study’s cardiologist and the patients’ GP, who then decided on changes in treatment. We believe that it is extremely unlikely that such an intervention might increase mortality. A close collaboration between nurses and physicians is essential in multidisciplinary care, which has shown to reduce the risk of HF hospitalization and mortality [5, 84] and is recommended by the ESC guidelines for the follow-up of persons with chronic HF [1]. However, a large-scale effectiveness study is clearly indicated to draw firm conclusions.

### Amendments to the intervention

Based on our results, we propose no significant amendments to the components of the intervention. However, activities within the components for delivery as well as for the preparatory education module need to be updated for relevant changes between the 2016 [2] and 2021 ESC [1] guidelines. For example, activities within our intervention’s component “medication review & optimization discussion” need to be updated in line with new treatment recommendations. Another example is that 2021 guidelines recommend careful evaluation of persistent signs of congestion before hospital discharge and an early follow-up visit at 1–2 weeks post-discharge. Thus, while the pre-discharge evaluation needs to be added, the early follow-up visit has already been included in our intervention.

### Limitations

This study has limitations. First, deviations from the intervention protocol occurred during the COVID-19 lock-down period for patients who received follow-up telephone calls instead of face-to-face visits. It was impossible to deliver key features of the intervention related to the person’s health status through phone calls. However, this may have translated into an underestimation of the effect of the intervention. Second, while persons with HF are at high risk for early readmission after hospital discharge [1, 85], the follow-up period of 3 months is too short to adequately estimate mortality. Furthermore, clinical outcomes were obtained from the hospital’s electronic medical records and declarations by the participants’ GPs. It is possible that more events occurred than what was assessed through these information sources. Third, our attrition rate for completing PRO measures was high, and having a complete set of PRO responses might have changed results. Finally, we used a single hospital; therefore, it was not possible to blind participants, intervention nurses, nor the cardiologist providing usual care. Also, intervention reports were uploaded to hospital records and provided to usual care practitioners. However, we consider the risk of contamination bias as minimal, since intervention nurses were not involved in usual care, and this is a novel intervention not delivered as part of nurses’ usual care.

### Implications of the study findings

Our findings suggest that the recruitment of HF persons with current or recent decompensation is highly demanding. To achieve sufficient numbers of participants, a multicentre study might be necessary for the conduction of a full-scale trial to evaluate the effectiveness of the intervention. Regarding the intervention itself, it was developed before the publication of the 2021 ESC guidelines [1]. A review is therefore indicated, as well as an update of the education requirements for nurses delivering the intervention. For all intervention components, fidelity strategies should be enhanced. Finally, a future large-scale trial would need a longer follow-up period to have adequate numbers of clinical events, and including a broader database including death registries would also be useful.

## Conclusions

The prevention of worsening HF is meaningful for patients [82] and healthcare professionals alike. The described intervention is promising in this regard. The pilot RCT presented in this article has helped to address key aspects of the feasibility and acceptability of the intervention, as well as allowing effect size estimates. It can therefore help ensure that future trials are well designed and sufficiently powered to prove a fair test of the intervention ([14], p 181), thereby fulfilling a key role of pilot and feasibility studies. The effectiveness of this intervention on patient-reported and clinical outcomes needs to be demonstrated.

## Supplementary Information

**Additional file 1.** CONSORT 2010 checklist of information to include when reporting a pilot or feasibility trial*.

**Additional file 2.** Use CONSERVE-CONSORT for completed trial reports and CONSERVE-SPIRIT for trial protocols.

## Data Availability

Data will be shared on reasonable request to the first author.

## Abbreviations

CG: Control group
DPI: Electronic patient records
ES: Effect size
GP: General practitioner
HF: Heart failure
IG: Intervention group
KCCQ: Kansas City Cardiomyopathy Questionnaire
LOS: Length of stay, days spent in hospital
NYHA: New York Heart Association
PI: Principal Investigator
PPI: Patient and Public Involvement
PRO: Patient-reported outcome
PROM: Patient-reported outcome measure
QoL: Quality of life
RCT: Randomized controlled trial
REDCap: Research Electronic Data Capture
SCHFI: Self-care of Heart Failure Index
TAPQ: Treatment Acceptability and Preferences Questionnaire

## References

[1] McDonagh TA, Metra M, Adamo M, Gardner RS, Baumbach A, Bohm M, Burri H, Butler J, Celutkiene J, Chioncel O, et al. 2021 ESC Guidelines for the diagnosis and treatment of acute and chronic heart failure. Eur Heart J. 2021;42:3599-726. 10.1093/eurheartj/ehab368 34447992

[2] Ponikowski P, Voors AA, Anker SD, Bueno H, Cleland JG, Coats AJ, Falk V, Gonzalez-Juanatey JR, Harjola VP, Jankowska EA (2016). 2016 ESC Guidelines for the diagnosis and treatment of acute and chronic heart failure: The Task Force for the diagnosis and treatment of acute and chronic heart failure of the European Society of Cardiology (ESC) developed with the special contribution of the Heart Failure Association (HFA) of the ESC. Eur Heart J.

[3] Conrad N, Judge A, Tran J, Mohseni H, Hedgecott D, Crespillo AP, Allison M, Hemingway H, Cleland JG, McMurray JJV (2018). Temporal trends and patterns in heart failure incidence: a population-based study of 4 million individuals. Lancet.

[4] van Riet EE, Hoes AW, Wagenaar KP, Limburg A, Landman MA, Rutten FH (2016). Epidemiology of heart failure: the prevalence of heart failure and ventricular dysfunction in older adults over time. A systematic review. Eur J Heart Fail.

[5] Takeda A, Martin N, Taylor RS, Taylor SJ (2019). Disease management interventions for heart failure. Cochrane Database Syst Rev.

[6] Takeda A, Taylor SJ, Taylor RS, Khan F, Krum H, Underwood M (2012). Clinical service organisation for heart failure. Cochrane Database Syst Rev.

[7] Jonkman NH, Westland H, Groenwold RH, Agren S, Atienza F, Blue L, Bruggink-Andre de la Porte PW, DeWalt DA, Hebert PL, Heisler M (2016). Do self-management interventions work in patients with heart failure? An individual patient data meta-analysis. Circulation.

[8] Jonkman NH, Schuurmans MJ, Jaarsma T, Shortridge-Baggett LM, Hoes AW, Trappenburg JC (2016). Self-management interventions: proposal and validation of a new operational definition. J Clin Epidemiol.

[9] Jonkman NH, Westland H, Groenwold RH, Agren S, Anguita M, Blue L, Bruggink-Andre de la Porte PW, DeWalt DA, Hebert PL, Heisler M (2016). What are effective program characteristics of self-management interventions in patients with heart failure? An individual patient data meta-analysis. J Card Fail.

[10] Jonkman NH, Groenwold RH, Trappenburg JC, Hoes AW, Schuurmans MJ. Complex self-management interventions in chronic disease unravelled: a review of lessons learned from an individual patient data meta-analysis. J Clin Epidemiol. 2017;83:48-56. 10.1016/j.jclinepi.2017.01.004 28126599

[11] Leventhal ME, Denhaerynck K, Brunner-La Rocca HP, Burnand B, Conca-Zeller A, Bernasconi AT, Mahrer-Imhof R, Froelicher ES, De Geest S (2011). Swiss Interdisciplinary Management Programme for Heart Failure (SWIM-HF): a randomised controlled trial study of an outpatient inter-professional management programme for heart failure patients in Switzerland. Swiss Med Wkly.

[12] Blauer C, Mahrer-Imhof R, Brunner-La Rocca H, Muller C, Eze G, Milbich I, Spirig R (2011). Development and implementation of a multidisciplinary nurse-led educational programme for inpatients with heart failure: the Basel-HF-Programme. Pflege.

[13] Campbell M, Fitzpatrick R, Haines A, Kinmonth AL, Sandercock P, Spiegelhalter D, Tyrer P (2000). Framework for design and evaluation of complex interventions to improve health. BMJ.

[14] Richards DA, Rahm Hallberg I (2015). Complex interventions in health: an overview of research methods.

[15] Campbell NC, Murray E, Darbyshire J, Emery J, Farmer A, Griffiths F, Guthrie B, Lester H, Wilson P, Kinmonth AL (2007). Designing and evaluating complex interventions to improve health care. BMJ.

[16] Craig P, Dieppe P, Macintyre S, Michie S, Nazareth I, Petticrew M (2013). Developing and evaluating complex interventions: the new medical research council guidance. Int J Nurs Stud.

[17] Craig P, Petticrew M (2013). Developing and evaluating complex interventions: reflections on the 2008 MRC guidance. Int J Nurs Stud.

[18] Skivington K, Matthews L, Simpson SA, Craig P, Baird J, Blazeby JM, Boyd KA, Craig N, French DP, McIntosh E (2021). A new framework for developing and evaluating complex interventions: update of medical research council guidance. BMJ.

[19] Dracup K, Moser DK, Pelter MM, Nesbitt TS, Southard J, Paul SM, Robinson S, Cooper LS (2014). Randomized, controlled trial to improve self-care in patients with heart failure living in rural areas. Circulation.

[20] Riegel B, Masterson Creber R, Hill J, Chittams J, Hoke L (2016). Effectiveness of motivational interviewing in decreasing hospital readmission in adults with heart failure and multimorbidity. Clin Nurs Res.

[21] Sokalski T, Hayden KA, Raffin Bouchal S, Singh P, King-Shier K (2020). Motivational interviewing and self-care practices in adult patients with heart failure: a systematic review and narrative synthesis. J Cardiovasc Nurs.

[22] Vellone E, Rebora P, Ausili D, Zeffiro V, Pucciarelli G, Caggianelli G, Masci S, Alvaro R, Riegel B (2020). Motivational interviewing to improve self-care in heart failure patients (MOTIVATE-HF): a randomized controlled trial. ESC Heart Fail.

[23] Schäfer-Keller P, Santos G, Pasche J, Verga M-E, Graf D, Strömberg A, Richards DA. Designing a self-care support intervention for individuals with heart failure using the Medical Research Council (MRC) framework for the development and evaluation of complex interventions. EuroHeartCare. Milano; 2019. https://urldefense.com/v3/__https:/people.hes-so.ch/en/profile/1832958079-petra-schafer-keller?type=direct&view=conferences__;!!NLFGqXoFfo8MMQ!osdC8zBm7_jc56KBoB6buDd0TK4HdfVUAfTEXPyAw4A0OFPCeBFb5lmcqqdGohXbTBtW0Y5WXMJ8M1jd9pGIKKM-Yc3VkVvxOk8iQw4I4gsa$.

[24] Santos G, Vasserot K, Villeneuve H, Raccanello O, Graf D, Aubort N, Vona M, Augereau C, Moses Passini C, Richards DA, et al. Development of a Nurse-led Clinic for people living with Heart Failure (CINACARD): qualitative evaluation of health care professionals’ perceived challenges. Researching Complex Interventions in Health: The State of the Art. Exeter; 2015. https://urldefense.com/v3/__https:/people.hes-so.ch/en/profile/1832958079-petra-schafer-keller?type=direct&view=conferences__;!!NLFGqXoFfo8MMQ!osdC8zBm7_jc56KBoB6buDd0TK4HdfVUAfTEXPyAw4A0OFPCeBFb5lmcqqdGohXbTBtW0Y5WXMJ8M1jd9pGIKKM-Yc3VkVvxOk8iQw4I4gsa$.

[25] Schafer-Keller P, Santos GC, Denhaerynck K, Graf D, Vasserot K, Richards DA, Stromberg A (2021). Self-care, symptom experience, needs, and past health-care utilization in individuals with heart failure: results of a cross-sectional study. Eur J Cardiovasc Nurs.

[26] Pasche J, Schäfer-Keller P. What is the role of heart failure nurses with post-diploma education in clinical practice? Results of a Swiss survey. Swiss Congress for Health Professions: 03.09.2018 2018. Zürich; 2018. https://urldefense.com/v3/__https:/people.hes-so.ch/en/profile/1832958079-petra-schafer-keller?type=direct&view=conferences__;!!NLFGqXoFfo8MMQ!osdC8zBm7_jc56KBoB6buDd0TK4HdfVUAfTEXPyAw4A0OFPCeBFb5lmcqqdGohXbTBtW0Y5WXMJ8M1jd9pGIKKM-Yc3VkVvxOk8iQw4I4gsa$.

[27] Eldridge SM, Chan CL, Campbell MJ, Bond CM, Hopewell S, Thabane L, Lancaster GA (2016). CONSORT 2010 statement: extension to randomised pilot and feasibility trials. Pilot Feasibility Stud.

[28] Orkin AM, Gill PJ, Ghersi D, Campbell L, Sugarman J, Emsley R, Steg PG, Weijer C, Simes J, Rombey T (2021). Guidelines for reporting trial protocols and completed trials modified due to the COVID-19 pandemic and other extenuating circumstances: The CONSERVE 2021 statement. JAMA.

[29] Patient education material Heart Failure. Patientenkit Herzinsuffizienz [German]. Kit de formation pour les insuffisants cardiaques [French]. Kit d'istruzione per pazienti con insufficienza cardiaca [Italian]. https://swissheart.ch/.

[30] Miller WR, Rollnick S (2002). Motivational interviewing. Preparing people for change.

[31] Riegel B, Dickson VV, Garcia LE, Masterson Creber R, Streur M. Mechanisms of change in self-care in adults with heart failure receiving a tailored, motivational interviewing intervention. Patient Educ Couns. 2016;100:283-8. 10.1016/j.pec.2016.08.030 PMC5318245 27599712

[32] Riegel B, Jaarsma T, Stromberg A (2012). A middle-range theory of self-care of chronic illness. ANS Adv Nurs Sci.

[33] Riegel B, Dickson VV, Faulkner KM (2016). The situation-specific theory of heart failure self-care: revised and updated. J Cardiovasc Nurs.

[34] Riegel B, Dickson VV (2008). A situation-specific theory of heart failure self-care. J Cardiovasc Nurs.

[35] Lorig K. Patient education. A practical approach. (3rd ed.). Thousand Oaks, California: Sage Publications, Inc; 2001.

[36] Riegel B, Lee CS, Dickson VV, Carlson B (2009). An update on the self-care of heart failure index. J Cardiovasc Nurs.

[37] Spertus JA, Jones PG (2015). Development and validation of a short version of the Kansas City cardiomyopathy questionnaire. Circ Cardiovasc Qual Outcomes.

[38] Green CP, Porter CB, Bresnahan DR, Spertus JA (2000). Development and evaluation of the Kansas City cardiomyopathy questionnaire: a new health status measure for heart failure. J Am Coll Cardiol.

[39] Kroenke K, Spitzer RL, Williams JB and Lowe B. The Patient Health Questionnaire Somatic, Anxiety, and Depressive Symptom Scales: a systematic review. Gen Hosp Psychiatry. 2010;32:345-59. 10.1016/j.genhosppsych.2010.03.006 20633738

[40] Gustafsson M, Alehagen U, Johansson P (2015). Pocket-sized ultrasound examination of fluid imbalance in patients with heart failure: a pilot and feasibility study of heart failure nurses without prior experience of ultrasonography. Eur J Cardiovasc Nurs.

[41] Swamy V, Brainin P, Biering-Sorensen T, Platz E (2019). Ability of non-physicians to perform and interpret lung ultrasound: a systematic review. Eur J Cardiovasc Nurs.

[42] Spertus JA, Jones PG, Sandhu AT, Arnold SV (2020). Interpreting the Kansas City cardiomyopathy questionnaire in Clinical Trials and Clinical Care: JACC state-of-the-art review. J Am Coll Cardiol.

[43] McCormack B, McCance T. Person-Centered Nursing. Theory and Practice. Chichester, West Sussex, United Kingdom: Wiley-Blackwell; 2010.

[44] Ha Dinh TT, Bonner A, Clark R, Ramsbotham J and Hines S. The effectiveness of the teach-back method on adherence and self-management in health education for people with chronic disease: a systematic review. JBI Database Syst Rev Implement Rep. 2016;14:210-47. 10.11124/jbisrir-2016-2296 26878928

[45] Hoffmann TC, Glasziou PP, Boutron I, Milne R, Perera R, Moher D, Altman DG, Barbour V, Macdonald H, Johnston M (2014). Better reporting of interventions: template for intervention description and replication (TIDieR) checklist and guide. BMJ.

[46] Moser DK, Dickson V, Jaarsma T, Lee C, Stromberg A, Riegel B (2012). Role of self-care in the patient with heart failure. Curr Cardiol Rep.

[47] Lee CS, Tkacs NC, Riegel B (2009). The influence of heart failure self-care on health outcomes: hypothetical cardioprotective mechanisms. J Cardiovasc Nurs.

[48] Richards DA (2015). Complex interventions and the amalgamation of marginal gains: a way forward for understanding and researching essential nursing care?. Int J Nurs Stud.

[49] Sidani S, Epstein DR, Bootzin RR, Moritz P, Miranda J (2009). Assessment of preferences for treatment: validation of a measure. Res Nurs Health.

[50] Cossette S, Belaid H, Heppell S, Mailhot T, Guertin MC (2016). Feasibility and acceptability of a nursing intervention with family caregiver on self-care among heart failure patients: a randomized pilot trial. Pilot Feasibility Stud.

[51] Sekhon M, Cartwright M, Francis JJ. Acceptability of healthcare interventions: an overview of reviews and development of a theoretical framework. BMC Health Serv Res. 2017;17(1):288. 10.1186/s12913-017-2031-8 PMC5267473 28126032

[52] Sekhon M, Cartwright M, Francis JJ. Acceptability of health care interventions: a theoretical framework and proposed research agenda. Br J Health Psychol. 2018;17;288. 10.1111/bjhp.12295 29453791

[53] Zannad F, Garcia AA, Anker SD, Armstrong PW, Calvo G, Cleland JG, Cohn JN, Dickstein K, Domanski MJ, Ekman I (2013). Clinical outcome endpoints in heart failure trials: a European Society of Cardiology Heart Failure Association consensus document. Eur J Heart Fail.

[54] Thompson LE, Bekelman DB, Allen LA, Peterson PN (2015). Patient-reported outcomes in heart failure: existing measures and future uses. Curr Heart Fail Rep.

[55] Vellone E, Riegel B, Cocchieri A, Barbaranelli C, D’Agostino F, Antonetti G, Glaser D, Alvaro R. Psychometric testing of the self-care of heart failure index version 6.2. Res Nurs Health. 2013;36(5):500–11. 10.1002/nur.21554 23832431

[56] Barbaranelli C, Lee CS, Vellone E, Riegel B (2014). Dimensionality and reliability of the self-care of heart failure index scales: further evidence from confirmatory factor analysis. Res Nurs Health.

[57] Hejjaji V, Tang Y, Coles T, Jones PG, Reeve BB, Mentz RJ, Spatz ES, Dunlay SM, Caldwell B, Saha A (2021). Psychometric evaluation of the Kansas City cardiomyopathy questionnaire in men and women with heart failure. Circ Heart Fail.

[58] EuroQol Research Foundation (2019). EQ-5D-5L user guide.

[59] Eldridge SM, Chan CL, Campbell MJ, Bond CM, Hopewell S, Thabane L, Lancaster GA, on behalf of the PAFS consensus group (2016). CONSORT 2010 statement: extension to randomised pilot and feasibility trials. BMJ.

[60] Corder GW, Foreman DI (2009). Nonparametric statistics for non-statisticians: a step-by-step approach.

[61] Verhestraeten C, Weijers G, Debleu D, Ciarka A, Goethals M, Droogmans S, Maris M (2020). Diagnosis, treatment, and follow-up of heart failure patients by general practitioners: a Delphi consensus statement. PLoS ONE.

[62] Smeets M, Vaes B, Aertgeerts B, Raat W, Penders J, Vercammen J, Droogne W, Mullens W, Janssens S. Impact of an extended audit on identifying heart failure patients in general practice: baseline results of the OSCAR-HF pilot study. ESC Heart Fail. 2020;7:3950-61. 10.1002/ehf2.12990 PMC7754725 32969599

[63] Stromberg A, Martensson J, Fridlund B, Levin LA, Karlsson JE, Dahlstrom U (2003). Nurse-led heart failure clinics improve survival and self-care behaviour in patients with heart failure: results from a prospective, randomised trial. Eur Heart J.

[64] van Riet EE, Hoes AW, Limburg A, Landman MA, van der Hoeven H, Rutten FH (2014). Prevalence of unrecognized heart failure in older persons with shortness of breath on exertion. Eur J Heart Fail.

[65] Kahn M, Grayson AD, Chaggar PS, Ng Kam Chuen MJ, Scott A, Hughes C, Campbell NG (2022). Primary care heart failure service identifies a missed cohort of heart failure patients with reduced ejection fraction. Eur Heart J.

[66] Mercieca-Bebber R, Palmer MJ, Brundage M, Calvert M, Stockler MR, King MT (2016). Design, implementation and reporting strategies to reduce the instance and impact of missing patient-reported outcome (PRO) data: a systematic review. BMJ Open.

[67] Crocker JC, Ricci-Cabello I, Parker A, Hirst JA, Chant A, Petit-Zeman S, Evans D, Rees S (2018). Impact of patient and public involvement on enrolment and retention in clinical trials: systematic review and meta-analysis. BMJ.

[68] Swiss academies of arts and sciences [Schweizerische Akademie der Medizinischen Wissenschaften]. Patienten und Angehörige beteiligen [German]. Patients' involvement, vol. 11. Swiss Academie of medical sciences, 2016, Bern, Switzerland. https://urldefense.com/v3/__https:/www.bing.com/ck/a?!&&p=8e117987430d0aa6JmltdHM9MTY4NzQ3ODQwMCZpZ3VpZD0yOWI0YjJhMi04YTI0LTY5NmQtMDM0NC1hMGVjOGI3ZjY4ZTEmaW5zaWQ9NTQxOA&ptn=3&hsh=3&fclid=29b4b2a2-8a24-696d-0344-a0ec8b7f68e1&psq=Patienten*und*Angeh**Arige*beteiligen&u=a1aHR0cHM6Ly93d3cuYXNzbS5jaC9kYW0vamNyOjc2MDczMDk2LTM5NWEtNDY0MC04ZDgzLWM2ODQ4MDE2NDA0Ny9iZXJpY2h0X3NhbXdfcGF0aWVudGVuX2JldGVpbGlnZW4ucGRmIzp-OnRleHQ9UGF0aWVudGVuYmV0ZWlsaWd1bmclMjAlMjhpbSUyMEVuZ2xpc2NoZW4lMjAlQzIlQUJwYXRpZW50JTIwZW5nYWdlbWVudCVDMiVCQiUyOSUyMGthbm4lMjBhbHMlMjB1bWZhc3NlbmRlcixFbnRzY2hlaWR1bmdzcHJvemVzc2UlMjBpbiUyMGRlciUyMEdlc3VuZGhlaXRzZ2VzZWxsLSUyMHNjaGFmdCUyMGVpbmJlem9nZW4lMjB3ZXJkZW4lMjAlNUIxMCU1RC4__;KyvDtis!!NLFGqXoFfo8MMQ!osdC8zBm7_jc56KBoB6buDd0TK4HdfVUAfTEXPyAw4A0OFPCeBFb5lmcqqdGohXbTBtW0Y5WXMJ8M1jd9pGIKKM-Yc3VkVvxOk8iQx7qozxB$. Accessed 20 June 2023.

[69] Swiss Clinical Trial Organisation. Fact Sheet: Patient and Public Involvement (PPI). 2021. https://urldefense.com/v3/__https:/www.bing.com/search?q=Swiss*Clinical*Trial*Organisation.*Fact*Sheet*3A*Patient*and*Public*Involvement*(PPI).*2021.*access*3A*sctofactsheet-ppi-en.pdf*(snf.ch)*2C*27.05.2022.&cvid=a1db1cef2f164d37a7aff41ea9551bbf&aqs=edge..69i57j69i11004.382j0j9&FORM=ANAB01&DAF0=1&PC=U531__;KysrKyslKysrKysrKyUrKyUr!!NLFGqXoFfo8MMQ!osdC8zBm7_jc56KBoB6buDd0TK4HdfVUAfTEXPyAw4A0OFPCeBFb5lmcqqdGohXbTBtW0Y5WXMJ8M1jd9pGIKKM-Yc3VkVvxOk8iQ0Wh_GiZ$. Accessed 27 May 2022.

[70] Tansella M, Thornicroft G (2009). Implementation science: understanding the translation of evidence into practice. Br J Psychiatry.

[71] Hasson H, Richards DA, Rahm Hallberg I (2015). Intervention fidelity in clinical trials. Complex interventions in health an overview of research methods.

[72] Bellg AJ, Borrelli B, Resnick B, Hecht J, Minicucci DS, Ory M, Ogedegbe G, Orwig D, Ernst D, Czajkowski S (2004). Enhancing treatment fidelity in health behavior change studies: best practices and recommendations from the NIH behavior change consortium. Health Psychol.

[73] Carroll C, Patterson M, Wood S, Booth A, Rick J, Balain S (2007). A conceptual framework for implementation fidelity. Implement Sci.

[74] Spillane V, Byrne MC, Byrne M, Leathem CS, O’Malley M, Cupples ME. Monitoring treatment fidelity in a randomized controlled trial of a complex intervention. J Adv Nurs. 2007;60(3):343–52. 10.1111/j.1365-2648.2007.04386.x 17908130

[75] Jiang Y, Koh KWL, Ramachandran HJ, Nguyen HD, Lim S, Tay YK, Shorey S, Wang W (2021). The effectiveness of a nurse-led home-based heart failure self-management programme (the HOM-HEMP) for patients with chronic heart failure: a three-arm stratified randomized controlled trial. Int J Nurs Stud.

[76] Jiang Y, Koh KWL, Ramachandran HJ, Tay YK, Wu VX, Shorey S, Wang W. Patients’ experiences of a nurse-led, home-based heart failure self-management program: findings from a qualitative process evaluation. J Med Internet Res. 2021;23(4):e28216. 10.2196/28216 PMC8114165 33904823

[77] Becker G, Kempf DE, Xander CJ, Momm F, Olschewski M, Blum HE (2010). Four minutes for a patient, twenty seconds for a relative - an observational study at a university hospital. BMC Health Serv Res.

[78] Lee CS, Moser DK, Lennie TA, Riegel B (2011). Event-free survival in adults with heart failure who engage in self-care management. Heart Lung.

[79] Lee CS, Bidwell JT, Paturzo M, Alvaro R, Cocchieri A, Jaarsma T, Stromberg A, Riegel B, Vellone E (2018). Patterns of self-care and clinical events in a cohort of adults with heart failure: 1 year follow-up. Heart Lung.

[80] Butler J, Braunwald E, Gheorghiade M (2014). Recognizing worsening chronic heart failure as an entity and an end point in clinical trials. JAMA.

[81] Bozkurt B, Coats AJS, Tsutsui H, Abdelhamid CM, Adamopoulos S, Albert N, Anker SD, Atherton J, Bohm M, Butler J (2021). Universal definition and classification of heart failure: a report of the Heart Failure Society of America, Heart Failure Association of the European Society of Cardiology, Japanese Heart Failure Society and Writing Committee of the Universal Definition of Heart Failure: Endorsed by the Canadian Heart Failure Society, Heart Failure Association of India, Cardiac Society of Australia and New Zealand, and Chinese Heart Failure Association. Eur J Heart Fail.

[82] Kraai IH, Vermeulen KM, Hillege HL, Jaarsma T (2018). “Not getting worse” a qualitative study of patients perceptions of treatment goals in patients with heart failure. Appl Nurs Res.

[83] Kelkar AA, Spertus J, Pang P, Pierson RF, Cody RJ, Pina IL, Hernandez A, Butler J (2016). Utility of patient-reported outcome instruments in heart failure. JACC Heart failure.

[84] Savarese G, Lund LH, Dahlstrom U, Stromberg A (2019). Nurse-led heart failure clinics are associated with reduced mortality but not heart failure hospitalization. J Am Heart Assoc.

[85] Cheema B, Ambrosy AP, Kaplan RM, Senni M, Fonarow GC, Chioncel O, Butler J, Gheorghiade M (2018). Lessons learned in acute heart failure. Eur J Heart Fail.

