# Supplementary material for: A multicomponent complex intervention for supportive follow-up of persons with chronic heart failure: a randomized controlled pilot study (the UTILE project)
Source: Pilot Feasibility Stud. 2023 Jun 27;9:106. doi: 10.1186/s40814-023-01338-7 (PMC10294419; doi:10.1186/s40814-023-01338-7)
Supplement: Supplementary file 2 — Additional file 2. Use CONSERVE-CONSORT for completed trial reports and CONSERVE-SPIRIT for trial protocols. [file 40814_2023_1338_MOESM2_ESM.pdf]

CONSERVE Checklists

Use CONSERVE-CONSORT for completed trial reports and CONSERVE-SPIRIT for trial protocols.

**CONSERVE-CONSORT Extension: [DATE]**					
**Item**	**Item Title**	**Description**			**Page No.**
I.	Extenuating Circumstances	Describe the circumstances and how they constitute extenuating circumstances.			3
II.	Important Modifications	a. Describe how the modifications are important modifications.			3; 10-11; 14; 20; 22
		b. Describe the impacts and mitigating strategies, including their rationale and implications for the trial.			3; 10-11; 14; 20; 22
		c. Provide a modification timeline.			n/a
III.	Responsible Parties	State who planned, reviewed and approved the modifications.			10
IV.	Interim data	If modifications were informed by trial data, describe how the interim data were used, including whether they were examined by study group, and whether the individuals reviewing the data were blinded to the treatment allocation.			n/a
**CONSORT Number and Item**		For each row, if important modifications occurred check “direct impact” and/or “mitigating strategy” and describe the changes in the trial manuscript or supplement. Check “no change” for items that are unaffected in the extenuating circumstance.			**Page No.**
		**No Change**	**Impact***	**Mitigating Strategy****	
1	Title and abstract	X			
2	Introduction	X			
3	Methods: Trial Design	X			
4	Methods: Participants			x	3
5	Methods: Interventions	X			14
6	Methods: Outcomes	X			
7	Methods: Sample Size	X			

8-10	Methods: Randomisation	X			
11	Methods: Blinding	X			
12	Methods: Statistical methods	x			
13	Results: Participant flow	x			
14	Results: Recruitment			x	11
15	Results: Baseline data	x			
16	Results: Numbers analysed	x			
	Results: Outcomes and estimation	x			11; 14
18	Results: Ancillary analyses	x			
19	Results: Harms	x			
20	Discussion: Limitations	x			20; 22
21	Discussion: Generalisability	x			
	Other information: Registration			x	2
24	Other information: Protocol	x			
25	Other information: Funding	x			

*Aspects of the trial that are directly affected or changed by the extenuating circumstance and are not under the control of investigators, sponsor or funder.
**Aspects of the trial that are modified by the study investigators, sponsor or funder to respond to the extenuating circumstance or manage the direct impacts on the trial.
